# A repository of protein abundance data of drug metabolizing enzymes and transporters for applications in physiologically based pharmacokinetic (PBPK) modelling and simulation

**DOI:** 10.1038/s41598-019-45778-9

**Published:** 2019-07-04

**Authors:** Mayur K. Ladumor, Aarzoo Thakur, Sheena Sharma, Aravind Rachapally, Sarang Mishra, Priyanka Bobe, V. Kameswara Rao, Praneetha Pammi, Hari Kangne, David Levi, Ankit Balhara, Sriram Ghandikota, Anupama Joshi, Vivek Nautiyal, Bhagwat Prasad, Saranjit Singh

**Affiliations:** 1 Department of Pharmaceutical Analysis, NIPER, S.A.S. Nagar, Punjab 160062 India; 2 Indian Institute of Technology Kharagpur, Kharagpur, West Bengal 721302 India; 3 Department of Pharmaceutics, University of Washington, Seattle, Washington 98195 USA

**Keywords:** Protein databases, Translational research, Computational science, Pharmaceutics, Statistical methods

## Abstract

Population factors such as age, gender, ethnicity, genotype and disease state can cause inter-individual variability in pharmacokinetic (PK) profile of drugs. Primarily, this variability arises from differences in abundance of drug metabolizing enzymes and transporters (DMET) among individuals and/or groups. Hence, availability of compiled data on abundance of DMET proteins in different populations can be useful for developing physiologically based pharmacokinetic (PBPK) models. The latter are routinely employed for prediction of PK profiles and drug interactions during drug development and in case of special populations, where clinical studies either are not feasible or have ethical concerns. Therefore, the main aim of this work was to develop a repository of literature-reported DMET abundance data in various human tissues, which included compilation of information on sample size, technique(s) involved, and the demographic factors. The collation of literature reported data revealed high inter-laboratory variability in abundance of DMET proteins. We carried out unbiased meta-analysis to obtain weighted mean and percent coefficient of variation (%CV) values. The obtained %CV values were then integrated into a PBPK model to highlight the variability in drug PK in healthy adults, taking lamotrigine as a model drug. The validated PBPK model was extrapolated to predict PK of lamotrigine in paediatric and hepatic impaired populations. This study thus exemplifies importance of the DMET protein abundance database, and use of determined values of weighted mean and %CV after meta-analysis in PBPK modelling for the prediction of PK of drugs in healthy and special populations.

## Introduction

Altered physiology and variable protein abundance of drug metabolizing enzyme and transporter (DMET) proteins in special populations (paediatrics, pregnant women and diseased patients) predispose them to safety or efficacy risks. This necessitates the need for clinical dose optimization of narrow therapeutic index drugs, especially in these categories. It is difficult to perform clinical trials in many populations because of several ethical and logistical reasons^1^. To fill this gap, *in vitro*-*in vivo* extrapolation (IVIVE)-linked physiologically based pharmacokinetic (PBPK) modelling has become a promising tool to predict drug pharmacokinetics (PK) in special populations, based on healthy adult data^2^. The regulatory agencies, such as U.S. Food and Drug Administration (US FDA), European Medicines Agency (EMA), and Pharmaceuticals and Medical Devices Agency (PMDA), now encourage pharmaceutical companies to use PBPK modelling for safer and efficient clinical drug development^3,4^.

However, PBPK models are data hungry and require physiological data, such as the abundance of DMET proteins. Particularly, IVIVE of drug disposition using PBPK modelling systems, e.g., GastroPlus, Simcyp and PK-Sim, can be refined further by use of the reported physiological DMET protein levels. Quantitative DMET protein information can be predicted using activity and mRNA data as surrogates. However, poor correlation between mRNA versus protein levels and non-selectivity of activity assays limit their use in IVIVE-PBPK modelling. In addition, cross-reactivity of antibodies across DME isoforms is a limitation of Western blotting technique. Therefore, the use of liquid chromatography-tandem mass spectrometry (LC-MS/MS) based quantitative proteomics, which is considered more precise, reliable and efficient, is rapidly emerging to establish inter-individual abundance of DMET proteins^5–7^. Additionally, availability of multiple organ banks or tissues from commercial sources requires a high-throughput technique such as LC-MS/MS proteomics for simultaneous DMET quantification. There are multiple benefits of such studies, which include: i) establishment of association of DMET abundance with age, sex, genotype, ethnicity and disease, and ii) prediction of *in vivo* PK profiles from *in vitro* data using PBPK modelling and simulation (M&S)^8–11^.

Unavailability of DMET proteomics data from large sample cohorts is a limitation in this field, which generally requires compilation and interpretation of data collected from different laboratories. However, extensive heterogeneity is observed when results of different laboratories are evaluated. These inter-laboratory variations are attributed to biological as well as methodological differences. True inter-individual variability in the samples due to demographic differences is covered under biological heterogeneity, whereas methodological heterogeneity mainly arises due to technique differences, e.g., quality of samples, analytical method variability, etc. The term, statistical heterogeneity, accounts for combined biological and methodological heterogeneity^12^. Such inter-laboratory heterogeneity is studied by meta-analysis of DMET abundance data through calculation of weighted mean and percent coefficient of variation (%CV) values^13–17^. With the availability of tissue samples and LC-MS/MS proteomics, more DMET data are available from last few years, which necessitates continuous updation of meta-analysis results.

We envisaged to compile all the available literature data on DMET protein abundances, and offer the repository to the users in the form of an Excel spreadsheet. The repository, so developed, was meant to be uploaded into an existing user-friendly open-access online QPrOmics database (http://qpromics.uw.edu/qpromics/data/) of the University of Washington, Seattle, USA. It was also envisioned to update meta-analysis of the individual DMET protein abundance data in the repository, especially of non-cytochrome P450 (non-CYP) enzymes, such as uridine 5′-diphospho-glucuronosyltransferases (UGTs), carboxylesterases (CESs) and flavin-containing monooxygenases (FMOs). The %CV values, ought to be calculated using three different methods, were planned to be integrated into a PBPK model for describing variability in PK of lamotrigine, a model drug, in the adult population. After validating the healthy adult lamotrigine PBPK model with multiple clinical PK data in literature, the model was intended to be extrapolated to paediatric and hepatic impaired (HI) populations, by integrating changes in protein abundance due to age and HI, respectively. All these expected activities were successfully accomplished. The details are discussed herein.

## Results

### Compilation of DMET protein abundance data

**Figure 1:**
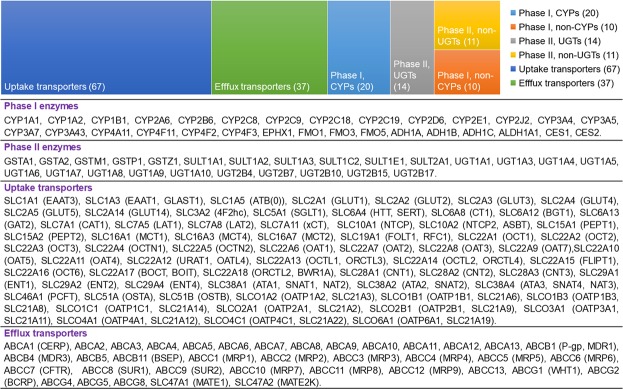
Number of drug metabolizing enzymes and transporters compiled and added into the repository (**a**) alongwith their common and alternate names (**b**).

We compiled literature reported protein abundance data of 55 DMEs (30 Phase I and 25 Phase II) and 104 membrane transporters (67 uptake and 37 efflux) (Fig. 1 and Supplementary Table S1). Wherever available, detailed demographic information, such as age, sex, ethnicity, genotype and disease conditions was also collated. The information in Supplementary Table S1 includes mean or median values of abundance, standard deviation (SD), range (minimum to maximum), %CV, abundance units, analytical method, relative or absolute quantification method, and references to the data source. The repository has been uploaded online as an open access user-friendly QPrOmics database at http://qpromics.uw.edu/qpromics and it is being updated regularly. The search from database is possible through either protein name, gene name or UniProt ID, which can be further refined by selecting the tissue of interest from various organs, viz., liver, intestine, kidney, brain and lungs. The search output information from website can be downloaded as an Excel spreadsheet (representative example shown in Supplementary Fig. S1).

### Assessment of heterogeneity in the abundance of non-CYP enzymes through meta-analyses

In the case of three non-CYP enzymes, viz., UGT1A4, UGT2B7 and CES1, the heterogeneity constant (H^2^) value was more than 1.5 in the case of fixed effect (FE) model and the same was below 1.2 in the random effect (RE) model. The H^2^ values more than 1.5 mean heterogeneity concern. Also, the FE model showed high to medium heterogeneity based on heterogeneity index (I^2^) data (Table 1). Considering this, we concluded that the inter-laboratory variability superseded true biological variability for these enzymes (Table 1). In UGT2B10, medium heterogeneity was observed for both FE and RE models. However, the result was shown to be statistically insignificant (P-value > 0.05) based on chi squared (χ^2^) distribution of the coefficient of heterogeneity (Q). The heterogeneity was low for all other enzymes (Table 1).

**Table 1:** Quantitative heterogeneity analysis of the analyzed hepatic non-CYP abundance data in liver microsomes. FE, RE and df represent fixed effect, random effect and degrees of freedom, respectively. Q_F_ and Q_R_ are coefficients of heterogeneity of FE and RE models, and H^2^ and I^2^ (%) are measures of heterogeneity. *represents the scenario where Q_F_ < df leading to an outcome of the RE model similar to the FE model.

Enzyme	df	FE model					RE model				
		Q_F_	H^2^	I^2^ (%)	P-value	Statistical Heterogeneity	Q_R_	H^2^	I^2^ (%)	P-value	Statistical Heterogeneity
UGT1A1	8	8.14	1.02	1.74	0.42	Low	7.92	0.99	0.00	0.44	None
UGT1A3*	5	2.56	0.51	0.00	0.77	None	2.56	0.51	0.00	0.77	None
UGT1A4	5	24.66	4.93	79.73	0.00016	High	3.23	0.65	0.00	0.66	None
UGT1A6	6	8.53	1.42	29.67	0.20	Low	7.50	1.25	19.97	0.28	Low
UGT1A9*	6	3.99	0.67	0.00	0.68	None	3.99	0.67	0.00	0.68	None
UGT2B4*	3	2.71	0.90	0.00	0.44	None	2.71	0.90	0.00	0.44	None
UGT2B7	6	31.24	5.21	80.79	0.000023	High	6.87	1.14	12.63	0.33	Low
UGT2B10	2	3.89	1.95	48.65	0.14	Medium	3.49	1.75	42.77	0.17	Medium
UGT2B15	5	6.61	1.32	24.33	0.25	Low	5.41	1.08	7.52	0.37	Low
UGT2B17*	3	1.27	0.42	0.00	0.74	None	1.27	0.42	0.00	0.74	None
CES1	1	2.41	2.41	58.45	0.12	Medium	1.00	1.00	0.00	0.32	None
FMO3*	2	1.27	0.63	0.00	0.53	None	1.27	0.63	0.00	0.53	None
FMO5*	1	0.18	0.18	0.00	0.67	None	0.18	0.18	0.00	0.67	None

### Determination of weighted mean and percent coefficient of variation values

The weighted mean values of abundance (pmol/mg microsomal protein) of non-CYP enzymes were determined to be in the following order: 1252.93 (CES1) > 75.21 (UGT2B7) > 49.43 (UGT2B4) > 46.77 (UGT1A4) > 41.50 (UGT2B15) > 35.97 (UGT1A1) > 29.71 (UGT1A6) > 29.33 (FMO3) > 27.38 (UGT1A9) > 25.01 (UGT1A3) > 24.63 (FMO5) > 14.72 (UGT2B10) > 5.84 (UGT2B17). The data for individual studies are shown in Supplementary Table S2.

**Figure 2:**
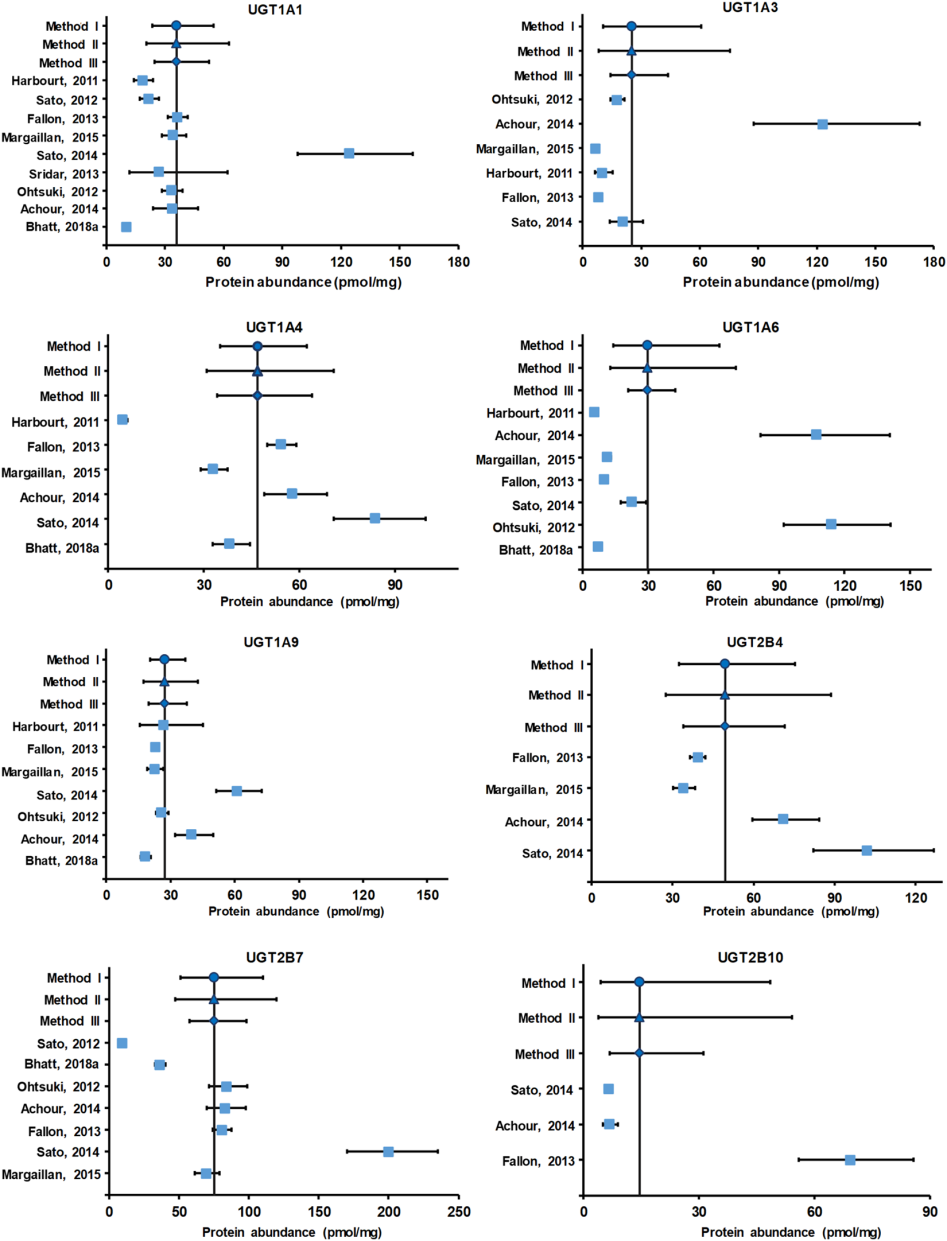
Forest plots representing hepatic protein abundance of UGT1A1, UGT1A3, UGT1A4, UGT1A6, UGT1A9, UGT2B4, UGT2B7 and UGT2B10. X-axis denotes protein abundance of enzyme in pmol/mg microsomal protein. Y-axis represents methods I-III and individual studies (first author name, year). Mean protein abundance value for the method I, method II, method III and individual studies are presented as filled circle, filled triangle, filled diamond and filled squares, respectively. The line perpendicular to X-axis denotes weighted mean, whereas lines parallel to X-axis denote 95% CI.

**Figure 3:**
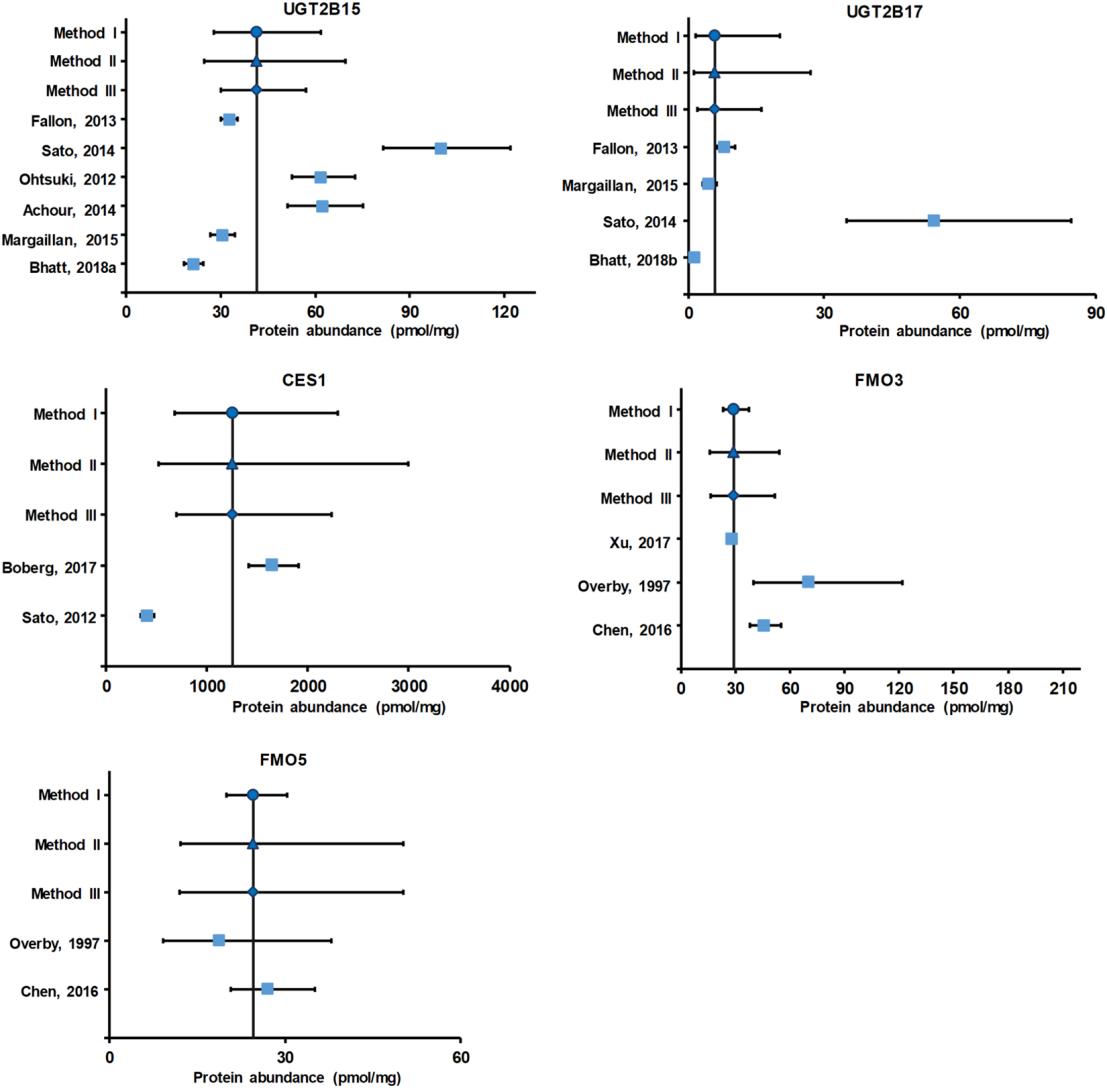
Forest plots representing hepatic protein abundance of UGT2B15, UGT2B17, CES1, FMO3 and FMO5. X-axis denotes protein abundance of enzyme in pmol/mg microsomal protein. Y-axis represents methods I-III and individual studies (first author name, year). Mean protein abundance value for the method I, method II, method III and individual studies are presented as filled circle, filled triangle, filled diamond and filled squares, respectively. The line perpendicular to X-axis denotes weighted mean, whereas lines parallel to X-axis denote 95% CI.

In general, the value of %CV or 95% confidence interval (CI), estimated using method II, was higher in comparison to methods I and III. Other observed advantage of method II was that it remained uninfluenced even when the studies were few in number. Also, it gave 95% CI range, which captured the observed variability. The meta-analyses results, including the weighted mean and %CV values for all non-CYP enzymes considered in the studies, are depicted in Table 2. The forest plots in Figs 2 and 3 provide a visual representation of the inter-laboratory variability across the mean for individual enzymes.

**Table 2:** Quantitative information based on pre-analyzed hepatic non-CYP abundance data in liver microsomes. %CV, CI, and k represent % coefficient of variation, confidence interval and number of studies, respectively.

Enzymes	Method type	Weighted mean (pmol/mg protein)	Weighted %CV	Weighted lower 95% CI	Weighted higher 95% CI	k
UGT1A1	I	35.97	72.36	23.54	54.97	9
	II		102.97	20.64	62.69	
	III		62.78	24.68	52.42	
UGT1A3	I	25.01	154.67	10.33	60.56	6
	II		238.29	8.30	75.33	
	III		78.45	14.37	43.53	
UGT1A4	I	46.77	36.96	35.12	62.27	6
	II		55.55	30.88	70.82	
	III		40.92	34.14	64.08	
UGT1A6	I	29.71	132.67	14.08	62.67	7
	II		169.39	12.55	70.32	
	III		50.58	20.86	42.31	
UGT1A9	I	27.38	41.72	20.35	36.84	7
	II		66.76	17.46	42.93	
	III		45.27	19.89	37.70	
UGT2B4	I	49.43	44.90	32.48	75.22	4
	II		65.21	27.58	88.58	
	III		39.24	34.11	71.62	
UGT2B7	I	75.21	55.48	51.24	110.40	7
	II		69.69	47.19	119.87	
	III		37.38	57.54	98.32	
UGT2B10	I	14.72	142.73	4.47	48.51	3
	II		166.74	3.99	54.28	
	III		74.75	6.93	31.28	
UGT2B15	I	41.50	52.90	27.89	61.75	6
	II		71.75	24.78	69.50	
	III		41.75	30.11	57.19	
UGT2B17	I	5.84	198.79	1.69	20.19	4
	II		324.38	1.26	27.05	
	III		138.85	2.12	16.14	
CES1	I	1252.93	45.92	683.36	2297.24	2
	II		69.68	523.92	2996.31	
	III		43.74	355.23	4419.19	
FMO3	I	29.33	21.58	23.03	37.34	3
	II		58.81	15.83	54.34	
	III		53.71	16.59	51.84	
FMO5	I	24.63	15.10	20.00	30.32	2
	II		54.98	12.08	50.21	
	III		55.08	12.07	50.27

### Prediction of the pharmacokinetics of lamotrigine in healthy adults and special populations

**Figure 4:**
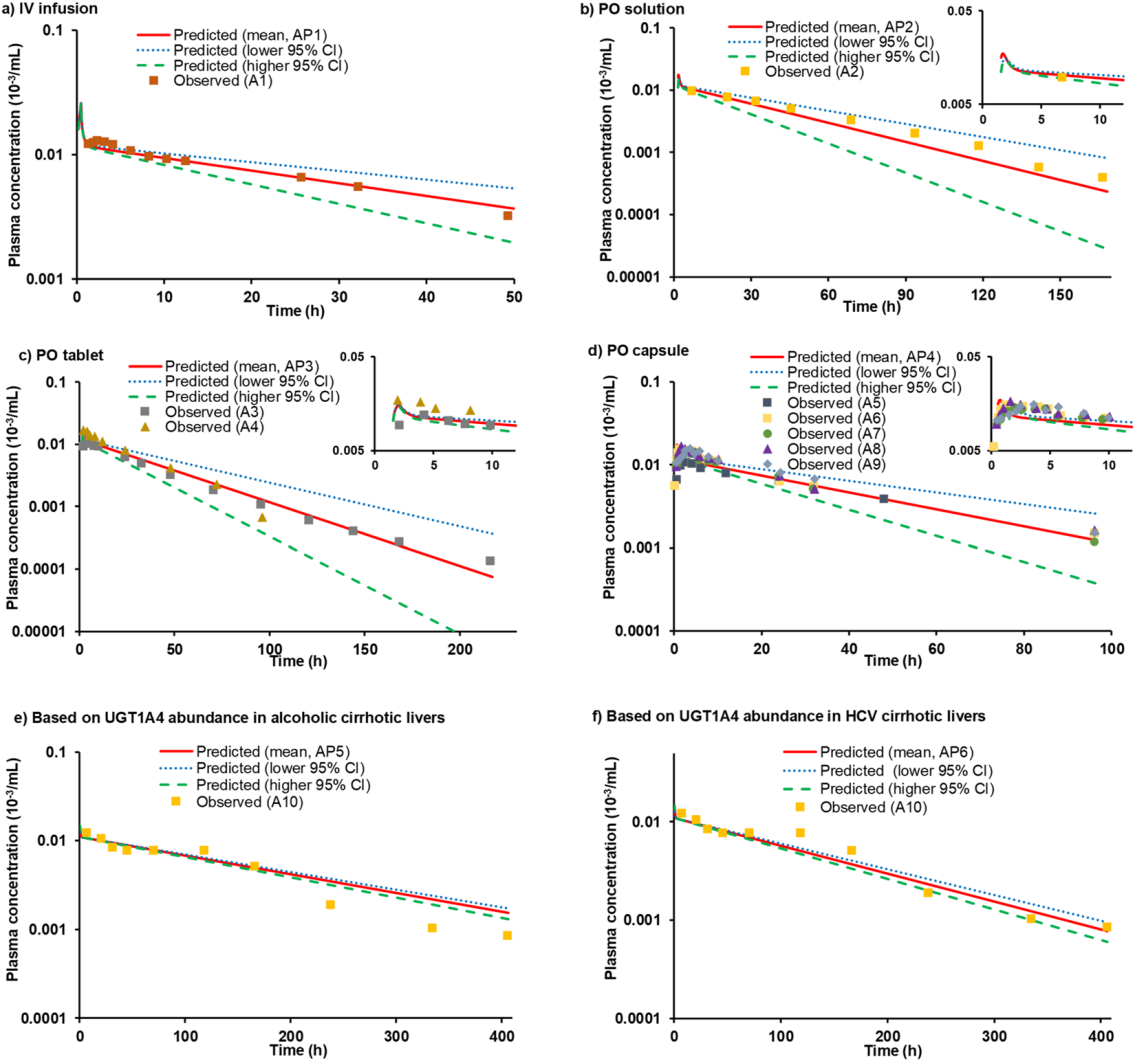
Observed versus predicted dose-normalized lamotrigine plasma concentration-time profiles after administration to adults of IV infusion (**a**), PO solution (**b**), PO tablet (**c**), and PO capsule (**d**). The profile for HI population are shown in (**e**) and (**f**). These profiles were generated by dividing the observed or predicted plasma concentrations by the dose. The symbols represent observed data, while the solid lines indicate the model predicted mean profile. The dotted and dashed lines represent the lower and higher 95% CI of protein abundances. Abbreviations used in the legends represent the following: AP1 (predicted mean, 67.8 mg IV infusion); A1 [observed, 67.8 mg^69^]; AP2 (predicted mean, 100 mg PO solution); A2 (observed, 100 mg^48^); AP3 (predicted mean, 200 mg PO tablet); A3 (observed, 200 mg^70^); A4 (observed, 100 mg^71^); AP4 (predicted mean, 120 mg PO capsule); A5 (observed, 25 mg^72^); A6 (observed, 30 mg^73^); A7 (observed, 60 mg^73^); A8 (observed, 120 mg^73^); A9 (observed, 240 mg^73^); AP5 (predicted mean, 100 mg PO solution); A10 (observed, 100 mg^48^), and AP6 (predicted mean, 100 mg). The liver cirrhosis data in Figures e and f are based on the abundance of UGT1A4 enzyme in alcoholic and HCV cirrhotic livers, respectively.

Figure 4 shows the predicted intravenous (IV) and peroral (PO) PK profiles of lamotrigine in healthy adults. The model was validated across various dosage forms (tablet, solution and capsule) and for an ascending dose of a capsule dosage form, and the resultant overlapping profiles are included in the same figure. The simulated lamotrigine plasma exposure parameters for both IV and PO studies were within the acceptance criteria (Table 3). Further, predicted results of lower and higher 95% CI values around the geometric weighted mean abundance, calculated using method II in adults, reasonably captured the variability in the observed data.

**Table 3:** Population acceptance criteria and predicted versus observed results for lamotrigine in healthy adult and special population after IV infusion and PO administration.

Study ID	Dose	Parameter	Mean observed (O)	Observed acceptance range	Mean predicted (P)	Predicted range	P/O ratio
**IV infusion (healthy adult)**							
A1	67.8 mg	C_max_	0.88	NA	1.76	1.74–1.77	2.00
A1	67.8 mg	AUC	31.33	NA	34.90	22.58–50.9	1.11
**PO solution (healthy adult)**							
A2	100 mg	C_max_	1.7	NA	1.54	1.51–1.56	0.91
A2	100 mg	AUC	66.5	NA	50.44	32.24–74.09	0.76
**PO tablet (healthy adult)**							
A3	200 mg	C_max_	2.46	1.99–3.04	3.05	2.98–3.09	1.24
A3	200 mg	AUC	94.6	70.82–126.37	101.17	64.73–148.48	1.07
A4	100 mg	C_max_	1.8	1.4–2.3	1.54	1.51–1.56	0.86
A4	100 mg	AUC	59.9	40.9–87.7	50.44	32.24–74.09	0.84
**PO capsule (healthy adult)**							
A5	25 mg	C_max_	0.29	0.22–0.39	0.38	0.37–0.39	1.32
A5	25 mg	AUC	11.73	7.24–19	12.58	8.03–18.49	1.07
A6	30 mg	C_max_	0.4	0.33–0.48	0.46	0.45–0.47	1.15
A6	30 mg	AUC	16.32	10.95–24.33	15.10	9.64–22.19	0.93
A7	60 mg	C_max_	0.8	0.73–0.88	0.92	0.9–0.93	1.15
A7	60 mg	AUC	33.96	27.40–42.09	30.23	19.31–44.42	0.89
A8	120 mg	C_max_	1.6	1.23–2.08	1.85	1.81–1.88	1.16
A8	120 mg	AUC	66	56.91–76.54	60.57	38.71–88.95	0.92
A9	240 mg	C_max_	3.16	2.26–4.42	3.63	3.54–3.68	1.15
A9	240 mg	AUC	152.2	120.24–192.65	121.54	77.92–178.36	0.80
**PO solution (HI adult, alcoholic cirrhosis liver)**							
A10	100 mg	C_max_	1.56	NA	1.29	1.28–1.29	0.83
A10	100 mg	AUC	197	NA	226.74	209.00–240.92	1.15
**PO solution (HI adult, hepatitis C cirrhosis liver)**							
A10	100 mg	C_max_	1.56	NA	1.29	1.28–1.29	0.83
A10	100 mg	AUC	197	NA	168.11	153.31–182.04	0.85
**PO tablet (early childhood)**							
P1	2 mg/kg	C_max_	1.1	0.55–2.21	1.83	1.82–1.84	1.66
P1	2 mg/kg	AUC	42.2	21.10–84.38	76.51	69.60–83.97	1.81
**PO tablet (middle childhood)**							
P2	2 mg/kg	C_max_	1.67	1.28–2.18	1.71	1.70–1.72	1.02
P2	2 mg/kg	AUC	70.4	31.25–158.60	90.10	80.43–99.29	1.28
**PO tablet (children)**							
P3	2 mg/kg	C_max_	1.48	1.05–2.08	1.75	4.54–4.55	1.18
P3	2 mg/kg	AUC	61	31.04–119.86	77.45	92.31–109.55	1.27

Observed and predicted maximum plasma concentration (C_max,_ μg/mL) and area under the plasma concentration-time profile (AUC, μg٠h/mL) are shown for the IV and PO routes of administration. Acceptance criteria were derived based on observed clinical C_max_ and AUC data. NA means not applicable (situation where %CV for C_max_ and AUC data were not available). Study ID: A1^69^, A2^48^, A3^70^, A4^71^, A5^72^, A6-A9^73^, A10^48^, P1-P3^47^.

The validated model was further extrapolated to predict PK in paediatric and HI populations, considering all the physiological changes including adjusted maximum velocity of the kinetic reaction (V_max_) and renal plasma clearance (CL_R_) values. The predicted results were within the range of observed clinical data (Table 3 and Fig. 4).

## Discussion

PBPK M&S is a main component of the model‐informed drug development (MIDD) and model‐informed precision dosing (MIPD)^18^. It finds application starting from first in human (FIH) dose selection, clinical study design onto dosing recommendations regarding drug interaction and pharmacogenetic effect in product labeling^4^. In that respect, M&S is targeted to reduction and/or replacement of human/animal studies^2,19^. Other benefit is that it facilitates benefit/risk assessment, whereby it enhances the likelihood of regulatory success. It is for these reasons that PBPK modelling is being currently widely encouraged even by the regulatory agencies, like US FDA^20^, EMA^21^ and PMDA^3^. For example, PBPK approaches have been included in regulatory guidance on drug-drug interactions (DDIs)^22–24^, paediatrics^25^, HI^26^, renal impairment (RI)^27^ and pharmacogenetics^28,29^ as a means to guide clinical study design and labeling decisions. Between 2008 and 2017, the FDA’s Office of Clinical Pharmacology (OCP) received 130 investigational new drug (IND) applications and 94 new drug applications (NDAs) containing PBPK analyses^30^. The utility of PBPK analyses in these regulatory submissions was primarily to assess enzyme-based DDIs (60%), followed by applications in paediatrics (15%), DDI with transporter (7%), HI (6%), RI (4%), absorption including food effect (4%), and pharmacogenetics (2%)^30^.

The successful application of PBPK modelling, which meets regulatory expectations, requires integration of drug-specific properties (molecular weight, pKa, logP, pH dependent solubility, apparent permeability, fraction of drug unbound in plasma (fu_p_), blood to plasma drug concentration ratio (B:P), etc.) with various physiology parameters (cardiac output, specific organ volume, tissue compositions, DMET abundance, transit times for luminal contents, etc.)^31^. With their help, the drug’s PK profile can be predicted and the same can be extrapolated across special populations, like paediatrics, pregnant women, maternal-fetal, HI and RI, etc.

Fortunately, drug specific properties such as solubility, permeability, enzyme and transport kinetics are extensively evaluated during early drug development. Further, for existing drugs, databases of drug specific properties are available in plenty, and some of them are freely accessible, like regulatory labels, DrugBank, etc. Good amount of information can also be accessed through material safety data sheets (MSDS).

The compilations and databases outlining body anatomy and physiology (e.g., tissue weights, blood flows to organs, tissue composition, etc.) in healthy and few special populations have been curated in the past several years. While individual physiology databases have been developed for Japanese^32,33^, Chinese^34^, and Indian populations^35^, a better geographically spread compilation is from 5-year effort conducted under the aegis of the International Atomic Energy Agency (IAEA), which accounted for the characteristics of populations in Bangladesh, China, India, Japan, Republic of Korea, Pakistan, Philippines, and Vietnam^36^. Similarly, National Center for Health Statistics (NCHS) designed a program, named National Health and Nutrition Examination Survey (NHANES), in order to assess the health and nutritional status of adults and children in the United States^37,38^. In the same way, Valentin compiled all the information on age- and gender-related differences in the anatomical and physiological characteristics of Western Europeans and North Americans, published earlier by the International Commission on Radiological Protection (ICRP) in 1975^39^. Another such attempt was made by Thompson *et al*. who compiled data from reported studies, including age-specific and clearance-related parameters in healthy and disease states^40^.

The clearance of drugs is primarily governed by DMET proteins, and hence the abundance of the latter has direct bearing on prediction of drug’s PK profiles and extrapolation to special populations. This is because abundance of DMET protein varies with demographic, biological and genetic factors, such as age, sex, ethnicity, disease condition and genotype^1^. This necessitates the availability of a repository containing quantitative information of DMET proteins. Therefore, the primary goal of the present study was to develop an online public repository that compiled the literature reported data on DMET proteins in various human tissues. Another target was to collate the information on the effect of associated covariates.

During the process of compiling the DMET abundance data, we observed vast inter-laboratory variability, which was higher than the anticipated biological variability^41^. This highlighted the need to derive more robust conclusions by performing meta-analyses, which provides good assessment of heterogeneity, and the calculated values of weighted mean and %CV, which can be integrated into PBPK modelling to predict the variability in PK^13–16,42^.

To assess heterogeneity as a part of meta-analyses, both FE and RE models were applied in the present study. Our results for UGT enzymes based on FE model (Table 1) were consistent with the previously published meta-analysis studies on same set of enzymes^14^. However, we observed that for two enzymes, where heterogeneity was evident through high I^2^ in the FE model, the RE model displayed none or low statistical heterogeneity. This indicated that FE model was perhaps a simpler model in describing statistical heterogeneity in this set of reported DME abundance data. A critical analysis of individual DME abundance studies showed major role of methodological heterogeneity, in the terms of sample source; its procurement and storage (frozen versus fresh tissue); sectioning procedure; sample preparation, and the technique of analysis. Among the latter, conventional immunoquantification-based methods like Western blotting, enzyme-linked immunosorbent assay (ELISA) and microarray can be considered less selective and low throughput than LC-MS/MS based quantification. However, inter-laboratory variability is also observed with LC-MS/MS proteomics, which can be attributed to the use of different peptides, differential protein extraction recovery, digestion efficiency and other methodological factors^5,41,43^. Therefore, harmonization of protein abundance determination protocol across laboratories is warranted.

The meta-analysis of UGT1A4 was in congruence with the large inter-individual variability in observed clinical PK of lamotrigine, which was successfully captured in the model by making use of %CV values of UGT1A4 protein abundance, obtained through method II (Fig. 4 and Table 3). The high inter-individual variability of this particular DME has been held responsible for adverse effects of lamotrigine, such as benign rashes, gastrointestinal disturbances and multi-organ failure associated with Steven Johnson syndrome^44,45^. In other reports, the reason for the observed variability has been attributed to underlying factors, such as age, gender, weight, co-medication, and state of renal and hepatic function^46–50^, highlighting the possibility of population effects.

Also, in the case of lamotrigine, therapeutic drug monitoring (TDM) is resorted for dose adjustment. Its serum concentration of 2.5–15 µg/mL is considered to be efficient and safe^51–53^. The drug is metabolized mainly by glucuronidation pathway, whose contribution is 86% and around 4% unidentified metabolites are formed^54^. The remaining 10% of drug dose is excreted unchanged in the urine^54^. The DMEs reported to be involved in lamotrigine metabolism are UGT1A3^46^, UGT1A4^46^ and UGT2B7^55^. However, more recent reports observed that UGT2B7 had no role in lamotrigine glucuronide formation^46^. Amongst UGT1A3 and UGT1A4, the latter is involved in ten-fold higher intrinsic clearance of the drug as compared to the former^46^. The neonatal level of UGT1A4 is ~50-fold lower than adult level^56^. Further, UGT1A4 abundance in alcoholic and hepatitis C virus (HCV) cirrhotic liver samples has been reported to be 12- and 4-fold lower than healthy liver samples, respectively^9^.

To describe UGT1A4 mediated variability in lamotrigine PK, we developed and validated a whole-body PBPK model of the drug using GastroPlus software. The predicted results were successfully able to capture, in particular, the elimination phase (Fig. 4), which is directly affected by the variability in UGT1A4. However, the absorption phase was not well captured. A high variability was also observed in lamotrigine absorption in clinic. The primary cause of the same remains to be ascertained.

For prediction of PK profile of lamotrigine in special population, age- and disease-dependent UGT1A4 abundance was integrated into the PBPK model, which well predicted the drug’s PK, even in the selected population. The differential protein abundance of UGT1A3 was not considered here, because of its small contribution to the metabolic clearance of lamotrigine and the lack of data. The extrapolated model well captured the PK parameters in children, including early and middle childhood and the obtained results were comparable to clinically reported PK values. In the case of HI population, while the PK parameters (AUC) were reasonably predicted, the simulated profile of lamotrigine was visually different than the observed clinical data^48^. This was perhaps due to the unknown etiology of the liver disease and its influence on the observed PK data reported in the clinical study. Moreover, the protein abundance of UGT1A4 is known to vary between alcoholic and HCV cirrhotic liver tissues^9^.

To summarize, a comprehensive repository of DMET protein abundance data was developed. Meta-analysis was successfully carried out on the compiled information to estimate overall variability (%CV) of protein abundance, and the influence of the latter on variability in PK profiles was established, taking lamotrigine as a model drug. The developed model was extrapolated to predict PK of lamotrigine in paediatric and HI populations.

## Methods

### *In silico* tools

Numerical values of abundance were extracted from the reported figures using GetData Graph Digitizer version 2.25 (http://getdata-graph-digitizer.com/). MySQL open source relational database management system (Cupertino, CA, USA) was used as a platform for the QPrOmics database. All the simulations for PBPK model development of lamotrigine were carried out in GastroPlus version 9.6 (Simulations Plus, Inc., Lancaster, California, USA). Figures for visual representation of statistical and simulation data were created using Excel 2016 (Microsoft, Redmond, WA); the same software was used for meta-analyses, and its in-build statistical function ‘CHIDIST’ was used to calculate P-values.

### Compilation of published DMET protein abundance data

Human DMET protein abundance data in different tissues with demographic details were from published literature that was searched through online search engines like PubMed, Google Scholar, Microsoft Academic, ScienceDirect, etc. For relevant search, the keyword combinations used were: drug metabolizing enzyme/transporter + abundance/expression + words like quantification/quantitation, LC-MS, LC-MS/MS, liquid chromatography-high resolution mass spectrometry (LC-HRMS), proteomics, or Western blotting/immunoblotting. Also the terms, such as quantity, concentration, content, quantification or measurement, were used as substitutes for the term “abundance/expression” to widen the search scope. The cross-references of individual articles were critically reviewed for any additional reported data. All available information till January 2019 for tissue distribution, donor demographics (including age, gender, ethnicity, genotype, disease, smoking, alcohol consumption and medication) and analytical methods employed were collated.

### Meta-analyses of protein abundance data of non-CYP enzymes

To demonstrate the utility of the database, a systematic meta-analysis was performed as per the Preferred Reporting Items for Systematic Reviews and Meta-Analyses (PRISMA) guideline^57^ to establish the overall abundance of non-CYP enzymes. The inclusion of reported data in meta-analyses was based on the following pre-defined criteria: i) considering only individual microsomal samples, excluding data from pooled donor samples; ii) taking absolute protein abundance values that were quantified by LC-MS/MS or Western blotting (immunoblotting), and excluding LC-MS global proteomics, mRNA expression levels and enzyme activity data, iii) including studies reporting data in picomole (pmol) per mg protein unit, but excluding studies with abundance data in arbitrary, relative or non-standard units; and iv) adding only those proteins where there were more than one reports from different laboratories.

Accordingly, for the purpose of this publication, only 18 out of 220 studies were included in the meta-analyses (Supplementary Fig. S2). The selected studies covered protein abundance data of the following non-CYP enzymes: UGT1A1, UGT1A3, UGTA4, UGT1A6, UGT1A9, UGT2B4, UGT2B7, UGT2B10, UGT2B15, UGT2B17, CES1, FMO3 and FMO5. The data were subjected to heterogeneity tests (weighting by inverse variance) to investigate intra- and inter-laboratory variability, which included determination of H^2^, I^2^, P-value and heterogeneity class^13,14,17,58^. Thereafter, the data were subjected to calculation of weighted mean (weighting by sample size)^13,14,17^, determination of %CV using three methods I–III. Eventually, 95% geometric CI, calculated using %CV (method II), was incorporated into the adult PBPK model of lamotrigine to explain the variability in its PK.

### Assessment of heterogeneity across studies

Summary estimates of studies and coefficient of heterogeneity were determined by FE and RE meta-analysis, to assess heterogeneity in data of different studies. The basic assumption for FE model is that the studies conducted are virtually identical (e.g., same study design, experimental conditions, etc.). On the other hand, the RE model assumes that the observed results may vary from study to study and follow certain distribution pattern^59^.

For FE model, summary estimate (*μ*_F_) of studies was calculated using Equation 1^13,17,58^.

1 $${\mu }_{{\rm{F}}}{\mathtt{=}}\sum _{{\rm{j}}{\mathtt{=}}{\mathtt{1}}}^{{\rm{k}}}{{\rm{w}}}_{{\rm{j}}}\cdot {{\rm{X}}}_{{\rm{j}}}{\mathtt{/}}\sum _{{\rm{j}}{\mathtt{=}}{\mathtt{1}}}^{{\rm{k}}}{{\rm{w}}}_{{\rm{j}}}$$

where, w_j_ is the FE weight of the study j, calculated as inverse of variance [w_j_ = 1/(SD_j_)^2^]; and X_j_ represents the mean abundance of a particular non-CYP enzyme for individual study j.

Further, Equation 2 was used to determine heterogeneity by FE model^58^.

2 $${{\rm{Q}}}_{{\rm{F}}}=\sum _{{\rm{j}}=1}^{{\rm{k}}}{{\rm{w}}}_{{\rm{j}}}\cdot {({{\rm{X}}}_{{\rm{j}}}-{{\rm{\mu }}}_{{\rm{F}}})}^{2}$$

where, Q_F_ [Cochran χ^2^-based Q test^60^] represents the coefficient of heterogeneity for the FE model. In RE model, summary estimate (*μ*_R_) was calculated using Equation 3.

3 $${{\rm{\mu }}}_{{\rm{R}}}=\sum _{{\rm{j}}=1}^{{\rm{k}}}{{\rm{w}}}_{{\rm{j}}}^{\ast }\cdot {{\rm{X}}}_{{\rm{j}}}/\sum _{{\rm{j}}=1}^{{\rm{k}}}{{\rm{w}}}_{{\rm{j}}}^{\ast }$$

where, ${{\rm{w}}}_{{\rm{j}}}^{\ast }$ is the RE weight of the study j, calculated by formula ${{\rm{w}}}_{{\rm{j}}}^{\ast }=1/({{\rm{w}}}_{{\rm{j}}}^{-1}+{\hat{{\rm{\tau }}}}^{2})$. Herein ${\hat{{\rm{\tau }}}}^{2}$ is the between-study heterogeneity estimator, which was obtained using Q_F_ and degrees of freedom (df; calculated as k-1, where k is the number of studies) by Equation 4^61^.

4 $${\hat{{\rm{\tau }}}}^{2}=\frac{{{\rm{Q}}}_{{\rm{F}}}-{\rm{df}}}{{\sum }_{{\rm{j}}=1}^{{\rm{k}}}{{\rm{w}}}_{{\rm{j}}}-(\frac{{\sum }_{{\rm{j}}=1}^{{\rm{k}}}{{{\rm{w}}}_{{\rm{j}}}}^{2}}{{\sum }_{{\rm{j}}=1}^{{\rm{k}}}{{\rm{w}}}_{{\rm{j}}}})}$$

The coefficient of heterogeneity of RE meta-analysis (Q_R_)^58^ was estimated when Q_F_ > df (Equation 5). Otherwise ${\hat{{\rm{\tau }}}}^{2}$ value was considered as zero, thus implying that RE meta-analysis would lead to same results as those obtained in FE meta-analysis.

5 $${{\rm{Q}}}_{{\rm{R}}}=\sum _{{\rm{j}}=1}^{{\rm{k}}}{{\rm{w}}}_{{\rm{j}}}^{\ast }\cdot {({{\rm{X}}}_{{\rm{j}}}-{{\rm{\mu }}}_{{\rm{R}}})}^{2}$$

Further, heterogeneity was determined through H^2^ and I^2^ indices for both FE and RE models using Equations 6 and 7, respectively.

6 $${{\rm{H}}}^{2}=\frac{{\rm{Q}}}{{\rm{df}}}$$

7 $${{\rm{I}}}^{2}( \% )=\frac{{{\rm{H}}}^{2}-1}{{{\rm{H}}}^{2}}\,\cdot 100$$

where, Q is coefficient of heterogeneity, defined as Q_F_ and Q_R_ for FE and RE models, respectively. As mentioned in discussion section, H^2^ values more than 1.5 generally cause considerable heterogeneity concern, while values below 1.2 are of little concern for heterogeneity^49^. The I^2^ index provides a percentage of overall variability between individual studies, with values 0%, ~25%, ~50% and ~75% classified as none, low, medium and high heterogeneity, respectively^13,14^. When I^2^ is negative, it is set to zero.

Further, P-values for determining the statistical significance of analysis were calculated using chi-squared (χ^2^) distribution of the Q and df values^13,14^.

### Calculation of weighted means and coefficient of variation

The meta-analysis was carried out considering weighting by sample size^13,17,62^. The weighted mean (WM) was calculated using Equation 8:

8 $${\rm{WM}}=\sum _{{\rm{j}}=1}^{{\rm{k}}}{{\rm{n}}}_{{\rm{j}}}\cdot {{\rm{X}}}_{{\rm{j}}}/{\rm{N}}$$

where, N is the number of samples in all studies $({\rm{N}}=\sum _{{\rm{j}}=1}^{{\rm{k}}}{{\rm{n}}}_{{\rm{j}}})$ and n_j_ is the number of samples in study j.

Amongst the three methods I-III used for calculation of %CV, variance (ν) and WM in method I were correlated as per Equation 9.

9 $$ \% \mathrm{CV}=\frac{{\rm{Overall}}\,{\rm{SD}}}{{\rm{WM}}}\cdot 100=\frac{\sqrt{{\rm{\nu }}}}{{\rm{WM}}}\cdot 100$$

where, *ν* was calculated^62^ in accordance with Equation 10:

10 $$\nu =\sum _{{\rm{j}}=1}^{{\rm{k}}}{{\rm{n}}}_{{\rm{j}}}\cdot {({{\rm{X}}}_{{\rm{j}}}-{\rm{WM}})}^{2}/{\rm{N}}$$

In method II, %CV was determined through overall SD and WM using Equation 11^63^.

11 $$ \% \mathrm{CV}=\frac{{\rm{Overall}}\,{\rm{SD}}}{{\rm{WM}}}\cdot 100=\frac{\sqrt{{\rm{Overall}}\,\mathrm{sum}\,\,{\rm{of}}\,{\rm{sqaures}}/{\rm{N}}}}{{\rm{WM}}}\cdot 100$$

where, the overall sum of squares was calculated considering standard deviation (SD_j_), X_j_ and n_j_ of individual study j, and WM employing Equation 12.

12 $${\rm{Overall}}\,{\rm{sum}}\,{\rm{of}}\,{\rm{squares}}=\sum _{{\rm{j}}=1}^{{\rm{k}}}\,[({({{\rm{SD}}}_{{\rm{j}}})}^{2}+{({{\rm{X}}}_{{\rm{j}}})}^{2})\cdot {{\rm{n}}}_{{\rm{j}}}]-{\rm{N}}\cdot {({\rm{WM}})}^{2}$$

In method III, %CV was calculated as weighting by sample size using Equation 13^13–15^:

13 $$ \% \mathrm{CV}=\frac{{\sum }_{{\rm{j}}=1}^{{\rm{k}}}{{\rm{n}}}_{{\rm{j}}}\cdot {{\rm{CV}}}_{{\rm{j}}}}{{\rm{N}}}\cdot 100$$

Lower and higher 95% CIs around the geometric weighted mean (WM) were calculated taking z value as 1.96 by using Equations 14 and 15^64^:

14 $${\rm{CI}}=\exp \,[\mathrm{ln}({\rm{WM}})\pm {\rm{z}}\cdot \frac{{\rm{\sigma }}}{\sqrt{{\rm{k}}}}]$$

15 $${\rm{\sigma }}=\sqrt{\mathrm{ln}\,[{(\frac{ \% \mathrm{CV}}{100})}^{2}+1]}$$

where, σ is the standard deviation of the data on the natural log scale.

### Development and validation of lamotrigine PBPK model and extension to special populations

#### Model development for healthy adult population

In the first step of PBPK model development for lamotrigine in GastroPlus, drug-specific physicochemical properties and system-specific input parameters were compiled, which are listed in Table 4.

**Table 4:** Input parameters for lamotrigine GastroPlus PBPK model.

	Parameters		Values/models	
Physiochemical and blood binding properties	Molecular weight (g/mol)		256.1^a^	
	LogP		1.19^b^	
	pK_a_		5.5^b^	
	Solubility (mg/mL) (pH = 7)		0.17^a^	
	B:P		1^b^	
	fu_p_		0.45^c^	
Absorption	Absorption model		ACAT^d^	
	P_eff_ (10^−4^ cm/sec)		7.761^c^	
	Diffusion coefficient (10^−5^ cm^2^/sec)		0.92^d^	
	Dissolution model		Johnson^d^	
	Particle size distribution		Log-normal^d^	
	Particle radius (µm)		25^d^	
	Particle density (g/mL)		1.2^d^	
	Dose volume (mL)		250^d^	
	Precipitation model		First order^d^	
	Precipitation time (sec)		900^d^	
	Paracellular model		Zhimin^d^	
Distribution	Distribution model		Full PBPK-Poulin & Theil (homogeneous)^d^	
	V_ss_ (L/kg)		1.16^c,e^	
Elimination	CL_IV_ (L/h)		2^c^	
	f_CL,renal_ (CL_R_ in L/h)		0.10 (0.2)^a^	
	f_CL,hepatic_ (CL_H_ in L/h)		0.90 (1.8)^a^	
Metabolic clearance	CLu_int,H_ in L/h		4.09^a^	
	f_m,UGT_ (CLu_int,UGT_ in L/h)		0.86 (3.91)^a^	
	f_m,CYP_ (CLu_int,CYP_ in L/h)		0.04 (0.18)^a^	
Extrapolation factor	ISEF		1^d^	
Fraction unbound	fu_mic_		1^d^	
Adult enzyme expression (mg enzyme/g tissue)	UGT1A3		0.016^d^	
	UGT1A4		0.017^d^	
**DME**_**j**_	**f**_**m,UGT**_ **(% contribution of DME**_**j**_ **)**	**K**_**m**_ **(µM)**	**V**_**max**_ **(pmol/min/pmol DME**_**j**_ **)** ^**f**^	${{\rm{C}}{\rm{L}}{\rm{u}}}_{{{\rm{i}}{\rm{n}}{\rm{t}}{\mathtt{,}}{\mathtt{\text{DME}}}}_{{\mathtt{j}}}}$ **(L/h)** ^**g**^
UGT1A3	0.086 (10)^h^	70^h^	0.96	0.39
UGT1A4	0.774 (90)^h^	550^h^	65.54	3.52

Abbreviations: LogP, partition coefficient; pK_a_, dissociation constant; B:P, blood to plasma concentration ratio; fu_p_, unbound fraction in the plasma; ACAT, advanced compartmental absorption and transit; P_eff_, effective permeability; V_ss_, volume of distribution at steady state; CL_IV_, intravenous plasma clearance; f_CL,renal_, fraction of drug cleared unchanged renally; CL_R_, renal plasma clearance; f_CL,hepatic_, fraction of drug cleared through hepatic metabolism; CL_H_, hepatic plasma clearance; CLu_int_,_H_, unbound hepatic intrinsic clearance; f_m,DME_, fraction of drug metabolized by a specific drug metabolizing enzyme isoform (DME_j_); ISEF, inter-system extrapolation factor; fu_mic_, unbound fraction in the microsomes; K_m_, concentration of substrate at which half-maximal enzymatic activity (V_max_) is reached, and CLu_int_, unbound intrinsic clearance for a particular metabolic pathway. For method/references, details are as follows: ^a^^54^, ^b^^49^, ^c^^65^, ^d^Default value in GastroPlus, ^e^Optimized according to literature reported^65^ value in adult by adjusting LogP value (1.629) with Poulin & Theil (homogeneous) method. Similar approach was used for V_ss_ calculation in paediatrics and HI populations, ^f^Calculated using Equation 19, ^g^Calculated using Equation 17, and ^h^^46^.

The next step involved development of a whole-body PBPK model for a healthy adult of 30 years age and 70 kg weight. The adult physiology was created using Population Estimates for Age Related (PEAR) physiology module within the simulator. In particular, intravenous plasma clearance (CL_IV_, L/h) and steady-state volume of distribution (V_ss_, L) values were taken from reported PBPK model of lamotrigine, which was primarily focused on optimal profiling of lamotrigine formulations, drug disposition and drug-drug interactions^65^. All tissues were assumed to be perfusion-limited compartments. Only liver tissue was considered for metabolic clearance of the drug.

#### *In vitro*-*in vivo* extrapolation (IVIVE)

The third step was development of a mechanistic model for which CL_IV_ and fraction of unchanged drug cleared through renal route (f_CL,renal_) data were used to estimate hepatic plasma clearance (CL_H_, 1.8 L/h) and CL_R_ (CL_R_ = f_CL,renal_ × CL_IV_, 0.2 L/h). The net unbound intrinsic hepatic clearance (CLu_int,H_, L/h) was back-calculated from CL_H_ by taking into account fu_p_ (0.45), and B:P (1) using “well-stirred” model^66^, as mentioned in Equation 16.

16 $${{\rm{C}}{\rm{L}}{\rm{u}}}_{{\rm{i}}{\rm{n}}{\rm{t}},{\rm{H}}}=\frac{{{\rm{Q}}}_{{\rm{H}},{\rm{B}}}\cdot {{\rm{C}}{\rm{L}}}_{{\rm{H}}}}{{{\rm{f}}{\rm{u}}}_{{\rm{p}}}\cdot ({{\rm{Q}}}_{{\rm{H}},{\rm{B}}}-{{\rm{C}}{\rm{L}}}_{{\rm{H}}}/{\rm{B}}:{\rm{P}})}$$

*In vivo* unbound intrinsic hepatic clearance for individual DME isoforms (${{\rm{C}}{\rm{L}}{\rm{u}}}_{{{\rm{i}}{\rm{n}}{\rm{t}},{\rm{D}}{\rm{M}}{\rm{E}}}_{{\mathtt{j}}}},{\mathtt{L}}{\mathtt{/}}{\mathtt{h}}$) was calculated using fraction metabolized by individual DME isoform (${{\rm{f}}}_{{{\mathtt{m}},{\rm{D}}{\rm{M}}{\rm{E}}}_{{\mathtt{j}}}}$), CLu_int,H_ values and the fraction of drug cleared through hepatic metabolism (f_CL,metabolism,H_ = 1 − f_CL,renal_) in accordance with Equation 17.

17 $${{\rm{C}}{\rm{L}}{\rm{u}}}_{{\rm{i}}{\rm{n}}{\rm{t}},{{\rm{D}}{\rm{M}}{\rm{E}}}_{{\rm{j}}}}=\,\frac{{{\rm{f}}}_{{\rm{m}},{{\rm{D}}{\rm{M}}{\rm{E}}}_{{\rm{j}}}}\cdot {{\rm{C}}{\rm{L}}{\rm{u}}}_{{\rm{i}}{\rm{n}}{\rm{t}},{\rm{H}}}}{{{\rm{f}}}_{{\rm{C}}{\rm{L}},{\rm{m}}{\rm{e}}{\rm{t}}{\rm{a}}{\rm{b}}{\rm{o}}{\rm{l}}{\rm{i}}{\rm{s}}{\rm{m}},{\rm{H}}}}$$

*In vitro* intrinsic hepatic clearance of individual DME isoforms (*in vitro* ${{\rm{C}}{\rm{L}}}_{{{\rm{i}}{\rm{n}}{\rm{t}},{\rm{D}}{\rm{M}}{\rm{E}}}_{{\mathtt{j}}}}$, µL/min/mg protein) was calculated using Equation 18.

18 $${\rm{I}}{\rm{n}}\,{\rm{v}}{\rm{i}}{\rm{t}}{\rm{r}}{\rm{o}}\,{{\rm{C}}{\rm{L}}}_{{\rm{i}}{\rm{n}}{\rm{t}},{{\rm{D}}{\rm{M}}{\rm{E}}}_{{\rm{j}}}}\,=\frac{{{\rm{C}}{\rm{L}}{\rm{u}}}_{{\rm{i}}{\rm{n}}{\rm{t}},{{\rm{D}}{\rm{M}}{\rm{E}}}_{{\rm{j}}}}}{{\rm{M}}{\rm{P}}{\rm{P}}{\rm{G}}{\rm{L}}\cdot {\rm{L}}{\rm{i}}{\rm{v}}{\rm{e}}{\rm{r}}\,{\rm{w}}{\rm{e}}{\rm{i}}{\rm{g}}{\rm{h}}{\rm{t}}\cdot 60\cdot {10}^{-6}}$$

where, MPPGL (mg of microsomal protein per g of liver; default GastroPlus value 38), liver weight (default GastroPlus value 1637.7 g) and a factor of 60 × 10^−6^ was used for unit conversion.

Thereafter, ${{\rm{V}}}_{{max,{\rm{D}}{\rm{M}}{\rm{E}}}_{{\mathtt{j}}}}$, which is maximum velocity of the kinetic reaction for individual DME isoforms (pmol/min/pmol isoform) was calculated using Equation 19.

19 $${{\rm{V}}}_{max,{{\rm{D}}{\rm{M}}{\rm{E}}}_{{\rm{j}}}}=\frac{{\rm{I}}{\rm{n}}\,{\rm{v}}{\rm{i}}{\rm{t}}{\rm{r}}{\rm{o}}\,{{\rm{C}}{\rm{L}}}_{{\rm{i}}{\rm{n}}{\rm{t}},{{\rm{D}}{\rm{M}}{\rm{E}}}_{{\rm{j}}}}\cdot {{\rm{K}}}_{{\rm{m}},{{\rm{D}}{\rm{M}}{\rm{E}}}_{{\rm{j}}}}\cdot {{\rm{f}}{\rm{u}}}_{{\rm{m}}{\rm{i}}{\rm{c}}}}{{{\rm{D}}{\rm{M}}{\rm{E}}}_{{\rm{j}}}\,{\rm{a}}{\rm{b}}{\rm{u}}{\rm{n}}{\rm{d}}{\rm{a}}{\rm{n}}{\rm{c}}{\rm{e}}\cdot {{\rm{I}}{\rm{S}}{\rm{E}}{\rm{F}}}_{{{\rm{D}}{\rm{M}}{\rm{E}}}_{{\rm{j}}}}}$$

where, ${{\mathtt{K}}}_{{{\mathtt{m}},{\rm{D}}{\rm{M}}{\rm{E}}}_{{\mathtt{j}}}}$ is the *in vitro* Michaelis-Menten constant (µM), fu_mic_ is the unbound fraction in microsomes (default GastroPlus value 1.0), DME_j _abundance is the abundance of individual DME isoforms in liver (default GastroPlus value 7.6 and 7.9 pmol/mg protein for UGT1A3 and UGT1A4, respectively), ${{\rm{ISEF}}}_{{{\rm{DME}}}_{{\rm{j}}}}$ is the inter-system extrapolation factor for individual DME isoforms (default GastroPlus value 1.0), as mentioned in Table 4.

To address inter-laboratory variability in specific protein abundance, we adjusted the ${{\mathtt{V}}}_{{max,{\mathtt{\text{DME}}}}_{{\mathtt{j}}}}$ values for individual enzymes using Equation 20 and then the same were input in the Enzymes and Transporter module of GastroPlus.

20 $${\rm{A}}{\rm{d}}{\rm{j}}{\rm{u}}{\rm{s}}{\rm{t}}{\rm{e}}{\rm{d}}\,{{\rm{V}}}_{max,{{\rm{D}}{\rm{M}}{\rm{E}}}_{{\rm{j}}}}{\mathtt{=}}{{\mathtt{V}}}_{max,{{\rm{D}}{\rm{M}}{\rm{E}}}_{{\rm{j}}}}\cdot {{\rm{S}}{\rm{F}}}_{{{\rm{C}}{\rm{I}}}_{{\rm{j}}}}$$

where, SF is the scale factor for inter-laboratory variability of specific DME, which was calculated using Equation 21.

21 $${{\rm{SF}}}_{{{\rm{CI}}}_{{\rm{j}}}}=\frac{{\rm{Weighted}}\,{\rm{lower}}\,{\rm{or}}\,{\rm{higher}}\,95 \% \,{\rm{CI}}\,{{\rm{DME}}}_{{\rm{j}}}\,{\rm{abundance}}\,{\rm{in}}\,{\rm{adults}}}{{\rm{Weighted}}\,{\rm{mean}}\,{{\rm{DME}}}_{{\rm{j}}}\,{\rm{abundance}}\,{\rm{in}}\,{\rm{adults}}}$$

The oral absorption model was established considering absorption parameters involving intestinal permeability, solubility, diffusion coefficient and particle size in “Human-Fasted” gut physiology model (default GastroPlus value except for permeability and solubility) with the disposition parameters as optimized above. Intestinal metabolism was assumed negligible as the oral bioavailability of lamotrigine is 98%.

#### Model evaluation

In a further step, the predictive performance of the developed models was evaluated by comparing the simulated exposure parameters with literature-based observed clinical exposure parameters (C_max_ and AUC), in accordance with the acceptance criteria suggested in the literature^64^. The lower and higher 99.998% CIs were calculated taking z value as 4.26 using Equations 14 and 15, and considering individual clinical PK data given in Supplementary Table S3.

#### Extrapolation of model to paediatric and HI population

The last step involved extrapolation of the validated adult PBPK model to predict PK in different paediatric populations, which was done using the PEAR Physiology module of GastroPlus. The models were developed for three age groups (representing average age and weight in each group), viz., early childhood (4 years, 17.34 kg), children (7 years, 26.54 kg) and middle childhood (9 years, 34.45 kg).

Although the protein abundance in adult, paediatric and HI population was considered similar in the software, to address protein abundance alteration, we adjusted the ${{\mathtt{V}}}_{{max,{\mathtt{\text{DME}}}}_{{\mathtt{j}}}}$ values for individual enzymes using Equation 22 and then the same were input in the Enzymes and Transporter module of GastroPlus.

22 $${\rm{Adjusted}}\,{{\rm{V}}}_{{\rm{\max }},{{\rm{DME}}}_{{\rm{j}}}}={{\rm{V}}}_{{\rm{\max }},{{\rm{DME}}}_{{\rm{j}}}}\cdot {{\rm{SF}}}_{{{\rm{DME}}}_{{\rm{j}}}}\cdot {{\rm{SF}}}_{{\rm{MPPGL}}}$$

where, SF is the scale factor, which was calculated as altered abundance of enzyme and MPPGL due to effect of age and disease (Supplementary Table S4), by using Equations 23 and 24, respectively:

23 $${{\rm{S}}{\rm{F}}}_{{{\rm{D}}{\rm{M}}{\rm{E}}}_{{\rm{j}}}}=\frac{\,{\rm{M}}{\rm{e}}{\rm{a}}{\rm{n}}\,{\rm{o}}{\rm{r}}\,95{\rm{ \% }}\,{\rm{C}}{\rm{I}}\,{{\rm{D}}{\rm{M}}{\rm{E}}}_{{\rm{j}}}\,{\rm{a}}{\rm{b}}{\rm{u}}{\rm{n}}{\rm{d}}{\rm{a}}{\rm{n}}{\rm{c}}{\rm{e}}\,{\rm{i}}{\rm{n}}\,{\rm{p}}{\rm{a}}{\rm{e}}{\rm{d}}{\rm{i}}{\rm{a}}{\rm{t}}{\rm{r}}{\rm{i}}{\rm{c}}/{\rm{H}}{\rm{I}}\,{\rm{p}}{\rm{o}}{\rm{p}}{\rm{u}}{\rm{l}}{\rm{a}}{\rm{t}}{\rm{i}}{\rm{o}}{\rm{n}}}{{\rm{M}}{\rm{e}}{\rm{a}}{\rm{n}}\,{{\rm{D}}{\rm{M}}{\rm{E}}}_{{\rm{j}}}\,{\rm{a}}{\rm{b}}{\rm{u}}{\rm{n}}{\rm{d}}{\rm{a}}{\rm{n}}{\rm{c}}{\rm{e}}\,{\rm{i}}{\rm{n}}\,{\rm{h}}{\rm{e}}{\rm{a}}{\rm{l}}{\rm{t}}{\rm{h}}{\rm{y}}\,{\rm{a}}{\rm{d}}{\rm{u}}{\rm{l}}{\rm{t}}{\rm{s}}}$$

24 $${{\rm{SF}}}_{{\rm{MPPGL}}}=\frac{{\rm{Mean}}\,{\rm{MPPGL}}\,{\rm{in}}\,{\rm{paediatric}}/{\rm{HI}}\,{\rm{population}}}{{\rm{Mean}}\,{\rm{MPPGL}}\,{\rm{in}}\,{\rm{healthy}}\,{\rm{adults}}}$$

The determinations were hence made for different age groups and HI population, especially for the abundance of UGT1A4^9,56^, and the results were incorporated into the model in order to capture differential PK of lamotrigine in these populations. Our published protein abundance data of UGT1A4 enzyme in alcoholic and HCV cirrhotic livers^9^ were used to develop two separate SFs for each of these populations. Further, scaled ${{\rm{C}}{\rm{L}}{\rm{u}}}_{{{\rm{i}}{\rm{n}}{\rm{t}},{\mathtt{\text{DME}}}}_{{\mathtt{j}}}}$ value was obtained through IVIVE using Equation 25:

25 $${\rm{S}}{\rm{c}}{\rm{a}}{\rm{l}}{\rm{e}}{\rm{d}}\,{{\rm{C}}{\rm{L}}{\rm{u}}}_{{\rm{i}}{\rm{n}}{\rm{t}},{{\rm{D}}{\rm{M}}{\rm{E}}}_{{\rm{j}}}}=\frac{{\rm{A}}{\rm{d}}{\rm{j}}{\rm{u}}{\rm{s}}{\rm{t}}{\rm{e}}{\rm{d}}\,{{\rm{V}}}_{max,{{\rm{D}}{\rm{M}}{\rm{E}}}_{{\rm{j}}}}\cdot {{\rm{D}}{\rm{M}}{\rm{E}}}_{{\rm{j}}}\,{\rm{a}}{\rm{b}}{\rm{u}}{\rm{n}}{\rm{d}}{\rm{a}}{\rm{n}}{\rm{c}}{\rm{e}}\cdot {{\rm{I}}{\rm{S}}{\rm{E}}{\rm{F}}}_{{{\rm{D}}{\rm{M}}{\rm{E}}}_{{\rm{j}}}}}{{{\rm{K}}}_{{\rm{m}},{{\rm{D}}{\rm{M}}{\rm{E}}}_{{\rm{j}}}}\cdot {{\rm{f}}{\rm{u}}}_{{\rm{m}}{\rm{i}}{\rm{c}}}}\cdot {\rm{M}}{\rm{P}}{\rm{P}}{\rm{G}}{\rm{L}}\cdot {\rm{L}}{\rm{i}}{\rm{v}}{\rm{e}}{\rm{r}}\,{\rm{w}}{\rm{e}}{\rm{i}}{\rm{g}}{\rm{h}}{\rm{t}}\cdot 60\cdot {10}^{-6}$$

Default GastroPlus liver weight values for each age group were input in the above mentioned Equation 25, which accounts for age-dependent change, viz., early childhood (592.12 g at 4 years), children (726.23 g at 7 years), middle childhood (906.24 g at 9 years). For Child-Pugh C class of HI population, liver weight taken was 867.97 g for 30 years age.

The renal plasma clearance in paediatric and HI group (CL_R,paediatric/HI_) were calculated^67^ using SF_fu×GFR_ (Supplementary Table S4), as described by Equations 26 and 27:

26 $${{\rm{CL}}}_{{\rm{R}},{\rm{paediatric}}/{\rm{HI}}}={{\rm{SF}}}_{{\rm{fu}}\times {\rm{GFR}}}\cdot {{\rm{CL}}}_{{\rm{R}}}$$

27 $${{\rm{SF}}}_{{\rm{fu}}\times {\rm{GFR}}}=\frac{{{\rm{fu}}}_{{\rm{paediatric}}/{\rm{HI}}}\cdot {{\rm{GFR}}}_{{\rm{paediatric}}/{\rm{HI}}}}{{{\rm{fu}}}_{{\rm{adult}}}\cdot {{\rm{GFR}}}_{{\rm{adult}}}}$$

where, fu_paediatric/HI_ and GFR_paediatric/HI_ values were obtained from GastroPlus.

The model was then simulated for oral formulations containing a dose of 2 mg/kg for early and middle childhood and children using in-built paediatric models. For all these paediatric populations, gastric emptying time (GET) value was set as 0.75 h^68^. The refined model was then used to predict PK in early and middle childhood and children populations, and the predictions were compared with the reported data using acceptance criteria as mentioned for the healthy adult population.

Similarly, the adult PBPK model was extrapolated to the diseased state model of HI population using PEAR Physiology module “Human, diseased, cirrhosis, Child-Pugh C”. The refined model was then used to predict PK in HI population and the predictions were compared with the reported observed data for the healthy adult population.

## Supplementary information

Supplementary information

Supplementary Table S1

## References

[1] Prasad B, Vrana M, Mehrotra A, Johnson K, Bhatt DK (2017). The promises of quantitative proteomics in precision medicine. J. Pharm. Sci..

[2] Hartmanshenn C, Scherholz M, Androulakis IP (2016). Physiologically-based pharmacokinetic models: approaches for enabling personalized medicine. J. Pharmacokinet. Pharmacodyn..

[3] Sato M (2017). Quantitative modeling and simulation in PMDA: a Japanese regulatory perspective. CPT Pharmacometrics Syst. Pharmacol..

[4] Shebley M (2018). Physiologically based pharmacokinetic model qualification and reporting procedures for regulatory submissions: a consortium perspective. Clin. Pharmacol. Ther..

[5] Bhatt DK, Prasad B (2018). Critical issues and optimized practices in quantification of protein abundance level to determine interindividual variability in DMET proteins by LC-MS/MS proteomics. Clin. Pharmacol. Ther..

[6] Ohtsuki S (2012). Simultaneous absolute protein quantification of transporters, cytochromes P450, and UDP-glucuronosyltransferases as a novel approach for the characterization of individual human liver: comparison with mRNA levels and activities. Drug Metab. Dispos..

[7] Xie C (2017). LC-MS/MS quantification of sulfotransferases is better than conventional immunogenic methods in determining human liver SULT activities: implication in precision medicine. Sci. Rep..

[8] Peng K-w, Bacon J, Zheng M, Guo Y, Wang MZ (2015). Ethnic variability in the expression of hepatic drug transporters: absolute quantification by an optimized targeted quantitative proteomic approach. Drug Metab. Dispos..

[9] Prasad B (2018). Abundance of phase I and II drug metabolizing enzymes in alcoholic and hepatitis C cirrhotic livers: a quantitative targeted proteomics study. Drug Metab. Dispos..

[10] Prasad B (2013). Interindividual variability in hepatic OATPs and P-glycoprotein (ABCB1) protein expression: quantification by LC-MS/MS and influence of genotype, age and sex. Drug Metab. Dispos..

[11] Zane NR, Chen Y, Wang MZ, Thakker DR (2018). Cytochrome P450 and flavin-containing monooxygenase families: age-dependent differences in expression and functional activity. Pediatr. Res..

[12] Rao G (2017). Methodological standards for meta-analyses and qualitative systematic reviews of cardiac prevention and treatment studies: a scientific statement from the American Heart Association. Circulation.

[13] Achour B, Barber J, Rostami-Hodjegan A (2014). Expression of hepatic drug-metabolizing cytochrome P450 enzymes and their inter-correlations: a meta-analysis. Drug Metab. Dispos..

[14] Achour B, Rostami‐Hodjegan A, Barber J (2014). Protein expression of various hepatic uridine 5′‐diphosphate glucuronosyltransferase (UGT) enzymes and their inter‐correlations: a meta‐analysis. Biopharm. Drug Dispos..

[15] Badée J, Achour B, Rostami-Hodjegan A, Galetin A (2015). Meta-analysis of expression of hepatic organic anion transporting polypeptide (OATP) transporters in cellular systems relative to human liver tissue. Drug Metab. Dispos..

[16] Burt HJ (2016). Abundance of hepatic transporters in Caucasians: a meta-analysis. Drug Metab. Dispos..

[17] Perrett H (2007). Disparity in holoprotein/apoprotein ratios of different standards used for immunoquantification of hepatic cytochrome P450 enzymes. Drug Metab. Dispos..

[18] Polasek Thomas M., Rayner Craig R., Peck Richard W., Rowland Andrew, Kimko Holly, Rostami‐Hodjegan Amin (2018). Toward Dynamic Prescribing Information: Codevelopment of Companion Model‐Informed Precision Dosing Tools in Drug Development. Clinical Pharmacology in Drug Development.

[19] Zhuang X, Lu C (2016). PBPK modeling and simulation in drug research and development. Acta Pharm. Sin. B.

[20] Center for Drug Evaluation and Research (CDER). Guidance for industry: physiologically based pharmacokinetic analyses-format and content, U.S. Food and Drug Administration, Silver Spring, MD (2018).

[21] Committee for Medicinal Products for Human Use (CHMP). Guideline on the reporting of physiologically based pharmacokinetic (PBPK) modelling and simulation, EMA/CHMP/458101/452016, European Medicines Agency, London (2018).

[22] Committee for Medicinal Products for Human Use (CHMP). Guideline on the investigation of drug interactions, CPMP/EWP/560/595/Rev. 561 Corr. 562**, European Medicines Agency, London (2012).

[23] Ministry of Health, Labour and Welfare Research Group. Drug interaction guideline for drug development and labeling recommendations (draft for public comment), Japanese Ministry of Health, Labour and Welfare, Tokyo (2014).

[24] Center for Drug Evaluation and Research (CDER). *In vitro* metabolism and transporter mediated drug-drug interaction studies, U.S. Food and Drug Administration, Silver Spring, MD (2017).

[25] Center for Drug Evaluation and Research (CDER). Guidance for industry: general clinical pharmacology considerations for pediatric studies for drugs and biological products, U.S. Food and Drug Administration, Silver Spring, MD (2014).

[26] Committee for Medicinal Products for Human Use (CHMP). Evaluation of the pharmacokinetics of medicinal products in patients with impaired hepatic function, CPMP/EWP/2339/2302, European Medicines Agency, London (2005).

[27] Committee for Medicinal Products for Human use (CHMP). Guideline on the evaluation of the pharmacokinetics of medicinal products in patients with decreased renal function, EMA/CHMP/83874/82014, European Medicines Agency, London (2015).

[28] Committee for Medicinal Products for Human Use (CHMP). Guideline on the use of pharmacogenetic methodologies in the pharmacokinetic evaluation of medicinal products, EMA/CHMP/37646/32009, European Medicines Agency, London (2011).

[29] Center for Drug Evaluation and Research (CDER). Guidance for industry: clinical pharmacogenomics: premarket evaluation in early-phase clinical studies and recommendations for labeling, U.S. Food and Drug Administration, Silver Spring, MD (2013).

[30] Grimstein M (2019). Physiologically based pharmacokinetic modeling in regulatory science: an update from the US Food and Drug Administration’s office of clinical pharmacology. J. Pharm. Sci..

[31] Kuepfer L (2016). Applied concepts in PBPK modeling: How to build a PBPK/PD model. CPT Pharmacometrics Syst. Pharmacol..

[32] Tanaka G-I, Kawamura H, Nakahara Y (1979). Reference Japanese man-I. Mass of organs and other characteristics of normal Japanese. Health phys..

[33] Tanaka G-I, Kawamura H, Nomura E (1981). Reference Japanese man-II. Distribution of strontium in the skeleton and in the mass of mineralized bone. Health Phys..

[34] Wang, J., Chen, R., Zhu, H., Zhou, Y. & Ma, R. Data of anatomical physiological and metabolic characteristics for Chinese reference man. 21–59 (Beijing: Atomic Energy Press, 1998).

[35] Jain S, Metha S, Kumar B, Reddy A, Nagaratnam A (1995). Formulation of the reference Indian adult: anatomic and physiologic data. Health phys..

[36] IAEA. Compilation of anatomical, physiological and metabolic characteristics for a reference asian man. Country reports 2 (1998).

[37] National Center for Health Statistics (NCHS). National Health and Nutrition Examination Survey (NHANES) https://www.cdc.gov/nchs/nhanes/about_nhanes.htm (2019).

[38] Price Paul S., Conolly Rory B., Chaisson Christine F., Gross Elizabeth A., Young John S., Mathis Eric T., Tedder Douglas R. (2003). Modeling Interindividual Variation in Physiological Factors Used in PBPK Models of Humans. Critical Reviews in Toxicology.

[39] Valentin J (2002). Basic anatomical and physiological data for use in radiological protection: reference values: ICRP Publication 89. Annals of the ICRP.

[40] Thompson CM (2009). Database for physiologically based pharmacokinetic (PBPK) modeling: Physiological data for healthy and health-impaired elderly. J. Toxicol. Environ. Health B Crit. Rev..

[41] Achour B (2017). Quantitative characterization of major hepatic UDP-glucuronosyltransferase (UGT) enzymes in human liver microsomes: comparison of two proteomic methods and correlation with catalytic activity. Drug Metab. Dispos..

[42] Chiba, K. *et al*. Prediction of inter-individual variability in pharmacokinetics of CYP2C19 substrates in humans. *Drug Metab. Pharmacokinet*, 379–386 (2014). 10.2133/dmpk.dmpk-13-rg-137 24739523

[43] Wegler C (2017). Variability in mass spectrometry-based quantification of clinically relevant drug transporters and drug metabolizing enzymes. Mol. Pharm..

[44] Hirsch L (2004). Correlating lamotrigine serum concentrations with tolerability in patients with epilepsy. Neurology.

[45] Kim SC, Kim MG (2019). Meta-analysis of the influence of UGT genetic polymorphisms on lamotrigine concentration. Basic Clin. Pharmacol. Toxicol..

[46] Argikar U, Remmel R (2009). Variation in glucuronidation of lamotrigine in human liver microsomes. Xenobiotica.

[47] Chen C, Casale EJ, Duncan B, Culverhouse EH, Gilman J (1999). Pharmacokinetics of lamotrigine in children in the absence of other antiepileptic drugs. Pharmacotherapy.

[48] Marcellin P (2001). Influence of cirrhosis on lamotrigine pharmacokinetics. Br. J. Clin. Pharmacol..

[49] Rambeck B, Wolf P (1993). Lamotrigine clinical pharmacokinetics. Clin. Pharmacokinet..

[50] Reimers A, Sjursen W, Helde G, Brodtkorb E (2016). Frequencies of UGT1A4*2 (P24T) and *3 (L48V) and their effects on serum concentrations of lamotrigine. Eur. J. Drug Metab. Pharmacokinet..

[51] Jacob S, Nair AB (2016). An updated overview on therapeutic drug monitoring of recent antiepileptic drugs. Drugs R. D..

[52] Johannessen SI, Tomson T (2006). Pharmacokinetic variability of newer antiepileptic drugs. Clin. Pharmacokinet..

[53] Perucca E (2000). Is there a role for therapeutic drug monitoring of new anticonvulsants?. Clin. Pharmacokinet..

[54] Center for Drug Evaluation and Research (CDER). Lamictal: lamotrigine tablets & chewable dispersible tablets, U.S. Food and Drug Administration, Silver Spring, MD (2003).

[55] Rowland A (2006). *In vitro* characterization of lamotrigine N2-glucuronidation and the lamotrigine-valproic acid interaction. Drug Metab. Dispos..

[56] Bhatt DK (2018). Age-and genotype-dependent variability in the protein abundance and activity of six major uridine diphosphate-glucuronosyltransferases in human liver. Clin. Pharmacol. Ther..

[57] Moher D, Liberati A, Tetzlaff J, Altman D (2009). Preferred reporting items for systematic reviews and meta-analyses: the PRISMA statement. Ann. Intern. Med..

[58] Higgins JP, Thompson SG (2002). Quantifying heterogeneity in a meta-analysis. Stat. Med..

[59] Riley RD, Higgins JP, Deeks JJ (2011). Interpretation of random effects meta-analyses. BMJ.

[60] Cochran WG (1954). The combination of estimates from different experiments. Biometrics.

[61] DerSimonian R, Laird N (1986). Meta-analysis in clinical trials. Contemp. Clin. Trials.

[62] Schmidt FL, Oh IS, Hayes TL (2009). Fixed- versus random-effects models in meta-analysis: model properties and an empirical comparison of differences in results. Br. J. Math. Stat. Psychol..

[63] Abduljalil, K., Johnson, T. N. & Rostami-Hodjegan, A. Fetal physiologically-based pharmacokinetic models: systems information on fetal biometry and gross composition. *Clin. Pharmacokinet*. **57**, 1149–1171 (2018). 10.1007/s40262-017-0618-1 29264787

[64] Abduljalil K, Cain T, Humphries H, Rostami-Hodjegan A (2014). Deciding on success criteria for predictability of pharmacokinetic parameters from *in vitro* studies: an analysis based on *in vivo* observations. Drug Metab. Dispos..

[65] Conner, T. M., Reed, R. C. & Zhang, T. A physiologically based pharmacokinetic model for optimally profiling lamotrigine disposition and drug-drug interactions. *Eur. J. Drug Metab. Pharmacokinet.* 1–20 (2018). 10.1007/s13318-018-0532-4 30460522

[66] Yang J, Jamei M, Yeo KR, Rostami-Hodjegan A, Tucker GT (2007). Misuse of the well-stirred model of hepatic drug clearance. Drug Metab. Dispos..

[67] Björkman S (2005). Prediction of drug disposition in infants and children by means of physiologically based pharmacokinetic (PBPK) modelling: theophylline and midazolam as model drugs. Br. J. Clin. Pharmacol..

[68] Johnson T, Bonner J, Tucker G, Turner D, Jamei M (2018). Development and applications of a physiologically-based model of paediatric oral drug absorption. Eur. J. Pharm. Sci..

[69] Peck A (1991). Clinical pharmacology of lamotrigine. Epilepsia.

[70] Wootton R (1997). Comparison of the pharmacokinetics of lamotrigine in patients with chronic renal failure and healthy volunteers. Br. J. Clin. Pharmacol..

[71] Keränen T, Sorri A, Moilanen E, Ylitalo P (2010). Effects of charcoal on the absorption and elimination of the antiepileptic drugs lamotrigine and oxcarbazepine. Arzneimittelforschung.

[72] Ebert U, Thong N, Oertel R, Kirch W (2000). Effects of rifampicin and cimetidine on pharmacokinetics and pharmacodynamics of lamotrigine in healthy subjects. Eur. J. Clin. Pharmacol..

[73] Cohen A (1987). Lamotrigine, a new anticonvulsant: pharmacokinetics in normal humans. Clin. Pharmacol. Ther..

