# Supplementary material for: A repository of protein abundance data of drug metabolizing enzymes and transporters for applications in physiologically based pharmacokinetic (PBPK) modelling and simulation
Source: Sci Rep. 2019 Jul 4;9:9709. doi: 10.1038/s41598-019-45778-9 (PMC6609630; doi:10.1038/s41598-019-45778-9)
Supplement: Supplementary file 1 — Supplementary information [file 41598_2019_45778_MOESM1_ESM.docx]

**A** **repository of protein abundance data of drug metabolizing enzymes and transporters for applications in physiologically based pharmacokinetic (PBPK) modelling and simulation**

Mayur K. Ladumor^1^, Aarzoo Thakur^1^, Sheena Sharma^1^, Aravind Rachapally^1^, Sarang Mishra^1^, Priyanka Bobe^1^, V. Kameswara Rao^1^, Praneetha Pammi^1^, Hari Kangne^1^, David Levi^1^, Ankit Balhara^1^, Sriram Ghandikota^1^, Anupama Joshi^1^, Vivek Nautiyal^2^, Bhagwat Prasad^3*^ & Saranjit Singh^1*^

^1^Department of Pharmaceutical Analysis, NIPER, S.A.S. Nagar, Punjab 160062, India.

^2^Indian Institute of Technology Kharagpur, Kharagpur, West Bengal 721302, India.

^3^Department of Pharmaceutics, University of Washington, Seattle, Washington 98195, USA.

*****Correspondence and requests for materials should be addressed to Saranjit Singh (email: [ssingh@niper.ac.in](mailto:ssingh@niper.ac.in)) and Bhagwat Prasad (email: bhagwat@uw.edu).

**
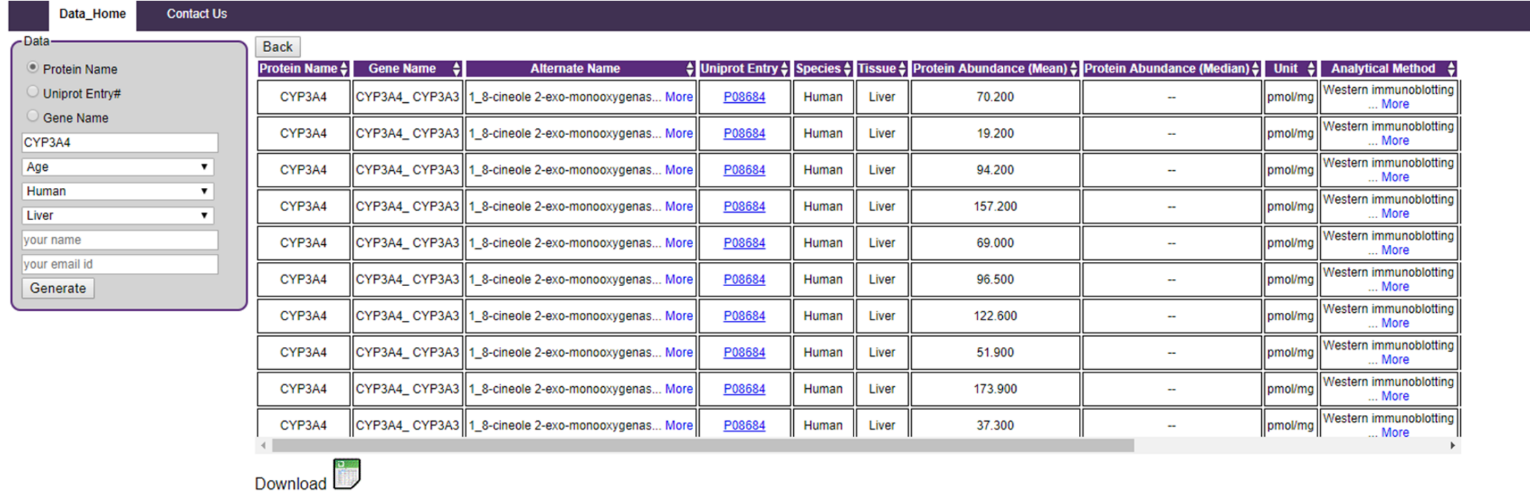
**

**Supplementary Figure S1. Screenshot of data module of QPrOmics database showing output for a representative protein search.**

**
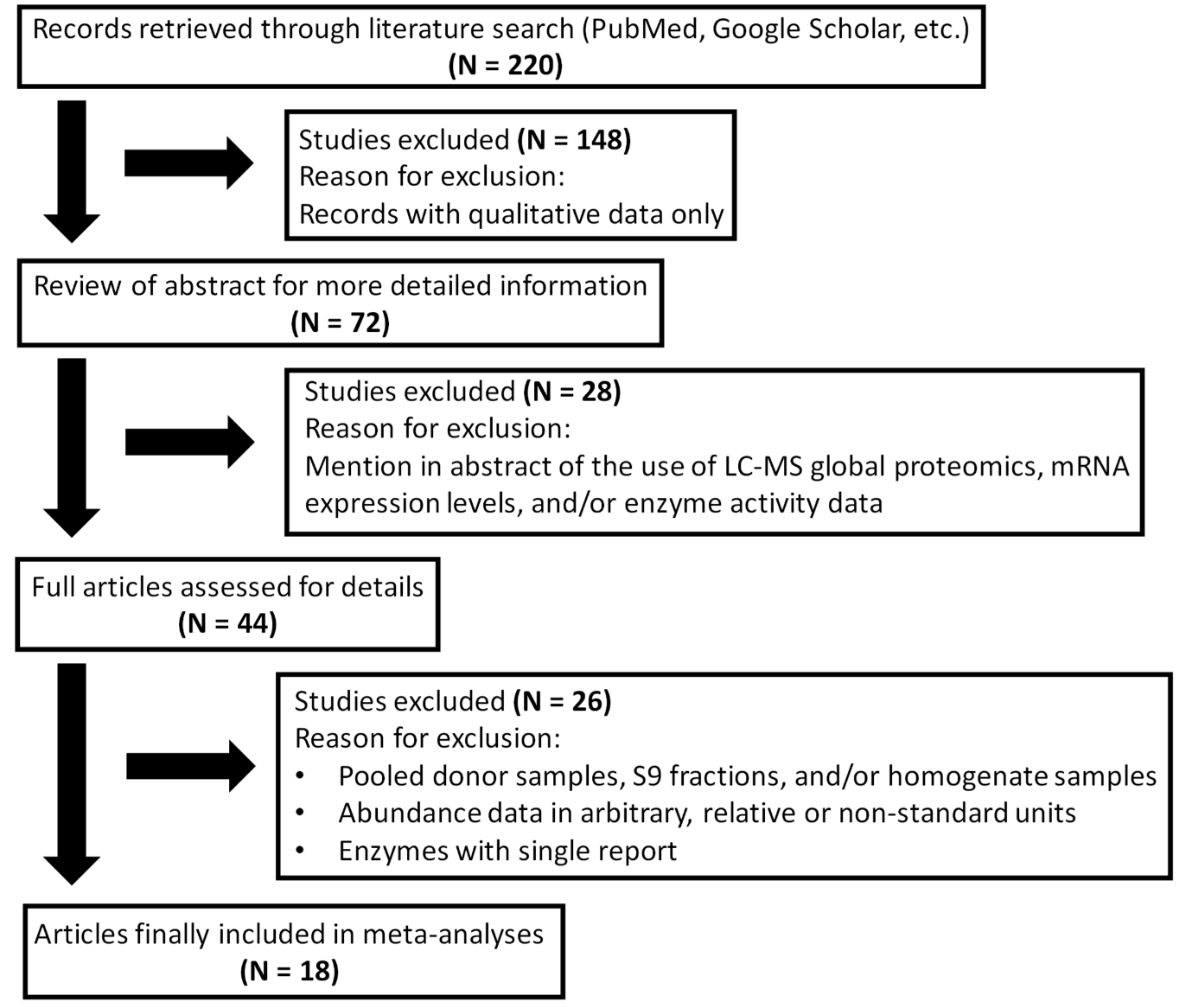
**

**Supplementary Figure S2. Study selection process followed before in the meta-analysis.**

**Supplementary Table S1. Quantitative information on abundance of DMET proteins (provided as an Excel file).**

|  | **Study name (First author, year)** | **Mean abundance**  **(pmol/mg protein)** | **Standard deviation (SD)** | **Number of samples (n)** | **% Coefficient of variation (%CV)** | **Sigma** | **Lower 95%CI** | **Higher 95%CI** | **References** |
| --- | --- | --- | --- | --- | --- | --- | --- | --- | --- |
| **UGT1A1** | Harbourt, 2011 | 18.3 | 7.8 | 9 | 42.62 | 0.41 | 10.24 | 32.69 | ^1^ |
|  | Sato, 2012 | 21.50 | 10.40 | 16 | 48.37 | 0.46 | 13.19 | 35.04 | ^2^ |
|  | Fallon, 2013 | 36.20 | 21.40 | 60 | 59.12 | 0.55 | 26.79 | 48.92 | ^3^ |
|  | Margaillan, 2015 | 34.00 | 24.82 | 48 | 73.00 | 0.65 | 22.75 | 50.82 | ^4^ |
|  | Sato, 2014 | 124.00 | 63.10 | 16 | 50.89 | 0.48 | 74.38 | 206.72 | ^5^ |
|  | Sridar, 2013 | 26.87 | 22.86 | 3 | 85.08 | 0.74 | 4.37 | 164.99 | ^6^ |
|  | Ohtsuki, 2012 | 33.20 | 11.50 | 17 | 34.64 | 0.34 | 23.45 | 47.01 | ^7^ |
|  | Achour, 2014 | 33.60 | 34.00 | 24 | 101.19 | 0.84 | 16.19 | 69.73 | ^8^ |
|  | Bhatt, 2018a | 10.00 | 5.70 | 31 | 57.00 | 0.53 | 6.66 | 15.01 | ^9^ |
| **UGT1A3** | Ohtsuki, 2012 | 17.30 | 7.80 | 17 | 45.09 | 0.43 | 11.09 | 26.98 | ^7^ |
|  | Achour, 2014 | 123.10 | 122.30 | 23 | 99.35 | 0.83 | 58.96 | 256.99 | ^8^ |
|  | [Margaillan, 2015](http://www.ncbi.nlm.nih.gov/pubmed/26076694) | 6.30 | 5.61 | 48 | 89.00 | 0.76 | 3.94 | 10.08 | ^4^ |
|  | Harbourt, 2011 | 9.90 | 7.40 | 9 | 74.75 | 0.67 | 3.84 | 25.50 | ^1^ |
|  | Fallon, 2013 | 8.00 | 5.30 | 60 | 66.25 | 0.60 | 5.74 | 11.15 | ^3^ |
|  | Sato, 2014 | 20.60 | 20.60 | 16 | 100.00 | 0.83 | 8.49 | 50.00 | ^5^ |
| **UGT1A4** | Harbourt, 2011 | 4.60 | 2.00 | 9 | 43.48 | 0.42 | 2.55 | 8.31 | ^1^ |
|  | Fallon, 2013 | 54.30 | 18.60 | 60 | 34.25 | 0.33 | 45.21 | 65.22 | ^3^ |
|  | [Margaillan, 2015](http://www.ncbi.nlm.nih.gov/pubmed/26076694) | 33.00 | 15.51 | 48 | 47.00 | 0.45 | 25.07 | 43.43 | ^4^ |
|  | Achour, 2014 | 58.00 | 24.80 | 23 | 42.76 | 0.41 | 40.30 | 83.47 | ^8^ |
|  | Sato, 2014 | 84.00 | 30.40 | 16 | 36.19 | 0.35 | 57.81 | 122.05 | ^5^ |
|  | Bhatt, 2018a | 38.20 | 17.10 | 31 | 44.76 | 0.43 | 27.55 | 52.98 | ^9^ |
| **UGT1A6** | Harbourt, 2011 | 5.20 | 2.30 | 9 | 44.23 | 0.42 | 2.85 | 9.48 | ^1^ |
|  | Achour, 2014 | 107.10 | 80.30 | 23 | 74.98 | 0.67 | 59.18 | 193.84 | ^8^ |
|  | Margaillan, 2015 | 11.30 | 5.88 | 48 | 52.00 | 0.49 | 8.36 | 15.27 | ^4^ |
|  | Fallon, 2013 | 9.70 | 4.20 | 60 | 43.30 | 0.41 | 7.72 | 12.18 | ^3^ |
|  | Sato, 2014 | 22.60 | 12.70 | 16 | 56.19 | 0.52 | 12.94 | 39.48 | ^5^ |
|  | Ohtsuki, 2012 | 114.00 | 54.00 | 17 | 47.37 | 0.45 | 71.62 | 181.46 | ^7^ |
|  | Bhatt, 2018a | 7.00 | 3.20 | 35 | 45.71 | 0.44 | 5.12 | 9.58 | ^9^ |
| **UGT1A9** | Harbourt, 2011 | 26.70 | 25.60 | 9 | 95.88 | 0.81 | 8.48 | 84.03 | ^1^ |
|  | Fallon, 2013 | 23.10 | 6.20 | 60 | 26.84 | 0.26 | 19.98 | 26.71 | ^3^ |
|  | Margaillan, 2015 | 22.60 | 14.69 | 48 | 65.00 | 0.59 | 15.69 | 32.56 | ^4^ |
|  | Sato, 2014 | 61.10 | 22.20 | 16 | 36.33 | 0.35 | 41.99 | 88.90 | ^5^ |
|  | Ohtsuki, 2012 | 25.90 | 6.30 | 17 | 24.32 | 0.24 | 20.22 | 33.18 | ^7^ |
|  | Achour, 2014 | 40.00 | 23.70 | 24 | 59.25 | 0.55 | 24.83 | 64.45 | ^8^ |
|  | Bhatt, 2018a | 18.10 | 7.50 | 35 | 41.44 | 0.40 | 13.59 | 24.11 | ^9^ |
| **UGT2B4** | Fallon, 2013 | 39.20 | 11.80 | 60 | 30.10 | 0.29 | 33.34 | 46.09 | ^3^ |
|  | Margaillan, 2015 | 34.00 | 15.30 | 48 | 45.00 | 0.43 | 26.11 | 44.27 | ^4^ |
|  | Achour, 2014 | 70.80 | 32.30 | 24 | 45.62 | 0.43 | 48.51 | 103.33 | ^8^ |
|  | Sato, 2014 | 102.00 | 47.60 | 16 | 46.67 | 0.44 | 63.58 | 163.65 | ^5^ |
| **UGT2B7** | Sato, 2012 | 9.67 | 3.68 | 16 | 38.06 | 0.37 | 6.54 | 14.31 | ^2^ |
|  | Bhatt, 2018a | 36.60 | 11.20 | 35 | 30.60 | 0.30 | 29.51 | 45.40 | ^9^ |
|  | Ohtsuki, 2012 | 84.30 | 29.50 | 17 | 34.99 | 0.34 | 59.34 | 119.77 | ^7^ |
|  | Achour, 2014 | 82.90 | 36.10 | 24 | 43.55 | 0.42 | 57.70 | 119.10 | ^8^ |
|  | Fallon, 2013 | 80.70 | 26.30 | 60 | 32.59 | 0.32 | 67.76 | 96.11 | ^3^ |
|  | Sato, 2014 | 200.00 | 67.80 | 16 | 33.90 | 0.33 | 140.76 | 284.17 | ^5^ |
|  | Margaillan, 2015 | 69.70 | 32.76 | 48 | 47.00 | 0.45 | 52.96 | 91.73 | ^4^ |
| **UGT2B10** | Sato, 2014 | 6.50 | 2.80 | 60 | 43.08 | 0.41 | 5.18 | 8.16 | ^5^ |
|  | Achour, 2014 | 6.80 | 8.43 | 48 | 124.00 | 0.96 | 3.76 | 12.31 | ^8^ |
|  | Fallon, 2013 | 69.30 | 31.70 | 16 | 45.74 | 0.44 | 43.56 | 110.24 | ^3^ |
| **UGT2B15** | Fallon, 2013 | 32.70 | 10.90 | 60 | 33.33 | 0.32 | 27.35 | 39.09 | ^3^ |
|  | Sato, 2014 | 99.70 | 42.60 | 16 | 42.73 | 0.41 | 64.46 | 154.20 | ^5^ |
|  | Ohtsuki, 2012 | 61.80 | 21.50 | 17 | 34.79 | 0.34 | 43.58 | 87.63 | ^7^ |
|  | Achour, 2014 | 62.10 | 31.50 | 24 | 50.72 | 0.48 | 40.96 | 94.15 | ^8^ |
|  | Margaillan, 2015 | 30.40 | 14.29 | 48 | 47.00 | 0.45 | 23.10 | 40.01 | ^4^ |
|  | Bhatt, 2018a | 21.20 | 9.70 | 35 | 45.75 | 0.44 | 15.49 | 29.02 | ^9^ |
| **UGT2B17** | Fallon, 2013 | 8.00 | 10.40 | 60 | 130.00 | 0.99 | 4.63 | 13.83 | ^3^ |
|  | Margaillan, 2015 | 4.40 | 8.10 | 48 | 184.00 | 1.22 | 2.08 | 9.29 | ^4^ |
|  | Sato, 2014 | 54.30 | 60.80 | 16 | 111.97 | 0.90 | 20.79 | 141.82 | ^5^ |
|  | Bhatt, 2018b | 1.33 | 1.76 | 185 | 132.33 | 1.01 | 0.97 | 1.82 | ^10^ |
| **CES1** | Boberg, 2017 | 1641.93 | 787.07 | 35 | 47.94 | 0.45 | 1183.40 | 2278.13 | ^11^ |
|  | Sato, 2012 | 402.00 | 139.00 | 16 | 34.58 | 0.34 | 281.06 | 574.99 | ^12^ |
| **FMO3** | Xu, 2017 | 27.90 | 15.23 | 228 | 54.59 | 0.51 | 24.16 | 32.22 | ^13^ |
|  | Overby, 1997 | 69.87 | 43.14 | 4 | 61.74 | 0.57 | 20.82 | 234.42 | ^14^ |
|  | Chen, 2016 | 45.63 | 13.95 | 10 | 30.58 | 0.30 | 30.50 | 68.26 | ^15^ |
| **FMO5** | Overby, 1997 | 18.75 | 15.47 | 4 | 82.51 | 0.72 | 4.04 | 87.01 | ^14^ |
|  | Chen, 2016 | 26.98 | 11.90 | 10 | 44.11 | 0.42 | 15.29 | 47.62 | ^15^ |

**Supplementary Table S2. Quantitative data for non-CYP enzymes in human liver microsomes, as reported in different studies and analysis of variation for each enzyme.**

| **Study number** | **Age in years (range)** | **Parameter** | **Dosage form** | **Dose (mg)** | **N** | **Mean** | **SEM** | **SD** | **%CV** | **Sigma** | **Higher 99.998% CI** | **Lower 99.998% CI** |
| --- | --- | --- | --- | --- | --- | --- | --- | --- | --- | --- | --- | --- |
|  |  |  |  |  |  |  |  |  |  |  |  |  |
| A1 | **-** | C_max_ | IV infusion | 67.82 | 8 | 0.88 | **-** | **-** | **-** | **-** | **-** | **-** |
| A1 | **-** | AUC | IV infusion | 67.82 | 8 | 31.33 | **-** | **-** | **-** | **-** | **-** | **-** |
| A2 | 50 | C_max_ | PO, solution | 100 | 12 | 1.7 | **-** | **-** | **-** | **-** | **-** | **-** |
| A2 | 50 | AUC | PO, solution | 100 | 12 | 66.5 | **-** | **-** | **-** | **-** | **-** | **-** |
| A3 | 35-57 | C_max_ | PO, tablet | 200 | 11 | 2.46 | - | 0.41 | 16.67 | 0.17 | 3.04 | 1.99 |
| A3 | 35-57 | AUC | PO, tablet | 200 | 11 | 94.6 | - | 21.6 | 22.83 | 0.23 | 126.37 | 70.82 |
| A4 | 22-27 | C_max_ | PO, tablet | 100 | 6 | 1.8 | - | 0.97 | 57.06 | 2.52 | 2.3 | 1.4 |
| A4 | 22-27 | AUC | PO, tablet | 100 | 6 | 59.9 | - | 51.9 | 86.6 | 0.75 | 87.7 | 40.9 |
| A5 | 25 | C_max_ | PO, capsule | 25 | 10 | 0.29 | 0.02 | 0.06 | 21.81 | 0.22 | 0.39 | 0.22 |
| A5 | 25 | AUC | PO, capsule | 25 | 10 | 11.73 | 1.37 | 4.34 | 36.97 | 0.36 | 19 | 7.24 |
| A6 | 36 | C_max_ | PO, capsule | 30 | 5 | 0.4 | - | 0.04 | 10 | 0.1 | 0.48 | 0.33 |
| A6 | 36 | AUC | PO, capsule | 30 | 5 | 16.32 | - | 3.46 | 21.2 | 0.21 | 24.33 | 10.95 |
| A7 | 36 | C_max_ | PO, capsule | 60 | 5 | 0.8 | - | 0.04 | 5 | 0.05 | 0.88 | 0.73 |
| A7 | 36 | AUC | PO, capsule | 60 | 5 | 33.96 | - | 3.84 | 11.31 | 0.11 | 42.09 | 27.4 |
| A8 | 36 | C_max_ | PO, capsule | 120 | 5 | 1.6 | - | 0.22 | 13.75 | 0.14 | 2.08 | 1.23 |
| A8 | 36 | AUC | PO, capsule | 120 | 5 | 66 | - | 5.14 | 7.79 | 0.08 | 76.54 | 56.91 |
| A9 | 36 | C_max_ | PO, capsule | 240 | 5 | 3.16 | - | 0.56 | 17.72 | 0.18 | 4.42 | 2.26 |
| A9 | 36 | AUC | PO, capsule | 240 | 5 | 152.2 | - | 18.9 | 12.42 | 0.12 | 192.65 | 120.24 |
| A10 | 53 | C_max_ | PO, solution | 100 | 5 | 1.56 | **-** | **-** | **-** | **-** | **-** | **-** |
| A10 | 53 | AUC | PO, solution | 100 | 5 | 197 | **-** | **-** | **-** | **-** | **-** | **-** |
| P1 | 4.8 | C_max_ | PO, tablet | 2 mg/kg | 4 | 1.1 | - | 0.37 | 33.64 | 0.33 | 2.21 | 0.55 |
| P1 | 4.8 | AUC | PO, tablet | 2 mg/kg | 4 | 42.2 | - | 14.1 | 33.41 | 0.33 | 84.38 | 21.1 |
| P2 | 9 | C_max_ | PO, tablet | 2 mg/kg | 8 | 1.67 | - | 0.3 | 17.96 | 0.18 | 2.18 | 1.28 |
| P2 | 9 | AUC | PO, tablet | 2 mg/kg | 8 | 70.4 | - | 40.9 | 58.1 | 0.54 | 158.6 | 31.25 |
| P3 | 7 | C_max_ | PO, tablet | 2 mg/kg | 12 | 1.48 | - | 0.42 | 28.38 | 0.28 | 2.08 | 1.05 |
| P3 | 7 | AUC | PO, tablet | 2 mg/kg | 12 | 61 | - | 36.2 | 59.34 | 0.55 | 119.86 | 31.04 |

**Supplementary Table S3. PK dataset with acceptance rage based on population criteria.** N**,** SEM, SD, %CV and sigma denotes number of subjects, standard error mean, standard deviation, percent coefficient of variation and geometric standard deviation, respectively. Observed C_max_ (μg/mL) and AUC (μg٠h/mL) are shown for the intravenous (IV) and oral (PO) routes of administration.

| **Population** | **SF_MPPGL_** | **SF_abundance,UGT1A4_** | | | **fu_p_** | **GFR (mL/min)** | $\text{SF}_{\text{fu}_{\text{p}}\text{×}\text{GFR}}$ | **CL_R_** |
| --- | --- | --- | --- | --- | --- | --- | --- | --- |
|  |  | **Mean abundance** | **Lower 95%CI abundance** | **Higher 95%CI abundance** |  |  |  |  |
| Neonates  (0 to 27 days) | 0.64 | 0.02 | 0.01 | 0.04 | 0.53 | 5.23 | 0.05 | 0.010 |
| Infants  (29 days to <2 years) | 0.65 | 0.17 | 0.06 | 0.44 | 0.49 | 23.70 | 0.21 | 0.043 |
| Early childhood  (1 to <6 years) | 0.70 | 0.56 | 0.37 | 0.84 | 0.47 | 55.49 | 0.48 | 0.097 |
| Middle childhood  (6 to <12 years) | 0.75 | 0.71 | 0.49 | 1.04 | 0.46 | 88.91 | 0.75 | 0.151 |
| Adult healthy  (>18 years) | 1.00 | 1.00 | 0.85 | 1.18 | 0.45 | 120.31 | 1.00 | 0.200 |
| Adult HI  (alcoholic cirrhotic)* | 1.00 | 0.08 | 0.04 | 0.18 | 0.58 | 88.91 | 0.96 | 0.192 |
| Adult HI  (HCV cirrhotic)* | 1.00 | 0.23 | 0.15 | 0.37 | 0.58 | 88.91 | 0.96 | 0.192 |

**Supplementary Table S4. Scale factors (SFs) values obtained for individual age groups.** Age classification based on the NICHD/NIH. *UGT1A4 abundance (in pmol/gram protein) is normalized with mg protein of gram of liver in HI (hepatic impaired) population. In case of adult healthy, SF_abundance,UGT1A3_ values: 0.33 (for lower 95% CI) and 3.01 (for higher 95% CI).

**References**

1 Harbourt, D. E. *et al.* Quantification of human uridine-diphosphate glucuronosyl transferase 1A isoforms in liver, intestine, and kidney using nanobore liquid chromatography-tandem mass spectrometry. *Anal. Chem.* **84**, 98-105 (2011).

2 Sato, Y., Nagata, M., Kawamura, A., Miyashita, A. & Usui, T. Protein quantification of UDP-glucuronosyltransferases 1A1 and 2B7 in human liver microsomes by LC-MS/MS and correlation with glucuronidation activities. *Xenobiotica* **42**, 823-829 (2012).

3 Fallon, J. K., Neubert, H., Hyland, R., Goosen, T. C. & Smith, P. C. Targeted quantitative proteomics for the analysis of 14 UGT1As and-2Bs in human liver using NanoUPLC-MS/MS with selected reaction monitoring. *J. Proteome Res.* **12**, 4402-4413 (2013).

4 Margaillan, G. *et al.* Multiplexed targeted quantitative proteomics predicts hepatic glucuronidation potential. *Drug Metab. Dispos.* **43**, 1331-1335 (2015).

5 Sato, Y. *et al.* Optimized methods for targeted peptide-based quantification of human uridine 5'-diphosphate-glucuronosyltransferases in biological specimens using liquid chromatography-tandem mass spectrometry. *Drug Metab. Dispos.* **42**, 885-889 (2014).

6 Sridar, C., Hanna, I. & Hollenberg, P. F. Quantitation of UGT1A1 in human liver microsomes using stable isotope-labelled peptides and mass spectrometry based proteomic approaches. *Xenobiotica* **43**, 336-345 (2013).

7 Ohtsuki, S. *et al.* Simultaneous absolute protein quantification of transporters, cytochromes P450, and UDP-glucuronosyltransferases as a novel approach for the characterization of individual human liver: comparison with mRNA levels and activities. *Drug Metab. Dispos.* **40**, 83-92 (2012).

8 Achour, B., Russell, M. R., Barber, J. & Rostami-Hodjegan, A. Simultaneous quantification of the abundance of several cytochrome P450 and uridine 5'-diphospho-glucuronosyltransferase enzymes in human liver microsomes using multiplexed targeted proteomics. *Drug Metab. Dispos.* **42**, 500-510 (2014).

9 Bhatt, D. K. *et al.* Age‐and genotype‐dependent variability in the protein abundance and activity of six major uridine diphosphate‐glucuronosyltransferases in human liver. *Clin. Pharmacol. Ther.* **105**, 131-141 (2018).

10 Bhatt, D. K. *et al.* Hepatic abundance and activity of androgen and drug metabolizing enzyme, UGT2B17, are associated with genotype, age, and sex. *Drug Metab. Dispos.* **46**, 888-896 (2018).

11 Boberg, M. *et al.* Age-dependent absolute abundance of hepatic carboxylesterases (CES1 and CES2) by LC-MS/MS proteomics: application to PBPK modeling of oseltamivir in vivo pharmacokinetics in infants. *Drug Metab. Dispos.* **45**, 216-223 (2017).

12 Sato, Y., Miyashita, A., Iwatsubo, T. & Usui, T. Simultaneous absolute protein quantification of carboxylesterases 1 and 2 in human liver tissue fractions using liquid chromatography tandem mass spectrometry. *Drug Metab. Dispos.* **40**, 1389-1396 (2012).

13 Xu, M. *et al.* Genetic and nongenetic factors associated with protein abundance of flavin-containing monooxygenase 3 in human liver. *J. Pharmacol. Exp. Ther.* **363**, 265-274 (2017).

14 Overby, L. H., Carver, G. C. & Philpot, R. M. Quantitation and kinetic properties of hepatic microsomal and recombinant flavin-containing monooxygenases 3 and 5 from humans. *Chem. Biol. Interact.* **106**, 29-45 (1997).

15 Chen, Y., Zane, N. R., Thakker, D. R. & Wang, M. Z. Quantification of flavin-containing monooxygenases 1, 3 and 5 in human liver microsomes by UPLC-MRM-based targeted quantitative proteomics and its application to the study of ontogeny. *Drug Metab. Dispos.* **44**, 975-983 (2016).
